# Universal response in the RKO colon cancer cell line to distinct antimitotic therapies

**DOI:** 10.1038/s41598-018-27267-7

**Published:** 2018-06-12

**Authors:** Alexander Lorz, Dana-Adriana Botesteanu, Doron Levy

**Affiliations:** 1 Computer, Electrical and Mathematical Sciences and Engineering Division, King Abdullah University of Science and Technology, Thuwal, Saudi Arabia; 2 Sorbonne Universités, Université Pierre et Marie Curie Université Paris 06, Unité mixte de recherche 7598, Laboratoire Jacques-Louis Lions, Paris, France; 3 Women’s Malignancies Branch, Center for Cancer Research, National Cancer Institute, National Institutes of Health, Bethesda, Maryland United States of America; 4 Department of Mathematics and Center for Scientific Computation and Mathematical Modeling, University of Maryland, College Park, Maryland United States of America; 5 Department of Discovery ADME, Boehringer Ingelheim RCV GmbH & Co KG, Vienna, Austria

## Abstract

Both classic and newer antimitotics commonly induce a prolonged mitotic arrest in cell culture. During arrest, cells predominantly undergo one of two fates: cell death by apoptosis, or mitotic slippage and survival. To refine this binary description, a quantitative understanding of these cell responses is needed. Herein, we propose a quantitative description of the kinetics of colon carcinoma RKO cell fates in response to different antimitotics, using data from the single cell experiments of Gascoigne and Taylor (2008). The mathematical model is calibrated using the *in vitro* experiments of Gascoigne and Taylor (2008). We show that the time-dependent probability of cell death or slippage is universally identical for monastrol, nocodazole and two different doses of AZ138, but significantly different for taxol. Death and slippage responses across drugs can be characterized by Gamma distributions. We demonstrate numerically that these rates increase with prolonged mitotic arrest. Our model demonstrates that RKO cells exhibit a triphasic response - first, remain in mitosis, then undergo fast and slow transition, respectively- dependent on the length of mitotic arrest and irrespective of cell fate, drug type or dose.

## Introduction

Classic microtubule-targeting drugs such as taxanes and vinca alkaloids constitute a highly successful class of antimitotic drugs, with potent anti-tumor activity in many human solid tumors^1–4^. In an effort to reduce the hematological and neuronal toxicity induced by these drugs and thus improve efficacy-to-toxicity ratios, newer antimitotic drugs such as spindle-targeting agents have been recently developed. However, these agents demonstrated limited anti-tumor activity in the clinic^5–12^. Despite their distinct primary targets, antimitotic drugs disrupt mitotic spindle assembly, activating the spindle assembly checkpoint (SAC), and leading to a prolonged mitotic arrest in 100% of the *in vitro* cells in the study irrespective of the antimitotic drug used^13^.

Following prolonged mitotic arrest, cancer cells predominantly undergo one of two fates: death in mitosis via intrinsic apoptosis, or slippage out of mitotic arrest following the gradual proteolysis of cyclin B1 and subsequent survival in an abnormal G1 state^14–17^. The proportion of cells that undergo each alternative fate and the timing of these events vary significantly between different drugs and cell types^7,13,14,18–23^. Even within identical types of cell cultures or drugs used, cells treated with antimitotics exhibit a considerable degree of heterogeneity in response to prolonged drug exposure^9,16,24^. Such observations have been reported in multiple single cell studies involving individual cancer cells in culture in the presence of various antimitotic drugs, including paclitaxel and Eg5 kinesin inhibitors.

Additionally, it has been experimentally demonstrated that even though the death in mitosis and mitotic slippage pathways are simultaneously active, they function independently of each other during mitotic arrest^18,25–28^. These studies confirmed Gascoigne and Taylor’s proposed “competing pathways model”, where the death in mitosis and mitotic slippage pathways are hypothesized to compete against each other (*i.e*., the fastest process to execute in an individual cell wins)^13^. The first pathway consists of the activation of cell death pathways, where caspase-dependent cell death signals become stronger in time, simultaneously as cyclin B1 degrades^13,15,16,18,24,26,29–34^. The second pathway involves cells that exit mitosis following a prolonged mitotic arrest, when cyclin B1 is slowly degraded and Cyclin-dependent kinase-1 (Cdk1) activity levels fall below the threshold needed to keep cells in mitosis and thus trigger mitotic exit, despite continued SAC signaling^2,16,25–27,32,34–37^. For example, in the case of Gascoigne and Taylor’s *in vitro* results on the colon carcinoma RKO cell line, the competing networks model would suggest that cell death signals in RKO cells accumulate faster than cyclin B1 levels degrade. Moreover, these accumulation rates would vary across cells, as implied by the different durations of mitotic arrest^13^.

The quantitative understanding of the cellular apoptosis and slippage rates and their dependency on the length of mitotic arrest is essential in order to decode and better understand the effect of the molecular mechanisms that govern cellular fate in response to antimitotic therapy. Furthermore, it remains to be elucidated whether any common features in the cellular responses to the different antimitotics characterizing each pathway exist.

In this paper, we propose a quantitative description of the kinetics of colon carcinoma RKO cells in response to the microtubule-targeting agents nocodazole and taxol, and the spindle-targeting Eg5 inhibitors AZ138 and monastrol. We hypothesize that the death in mitosis and mitotic slippage pathways exhibit differential cellular apoptosis and slippage rates depending on the length of mitotic arrest. Our mathematical model is calibrated using the *in vitro* observations of^13^, wherein time-lapse microscopy data demonstrated prolonged, variable durations of mitotic arrest in RKO cells prior to subsequent cell death or slippage.

Our aim is to provide a quantitative description of the RKO cellular apoptosis and slippage rates in response to distinct antimitotic drugs. By doing so, we report that RKO cells exhibit a triphasic response under prolonged exposure to the different antimitotics, *i.e*., first, remain in mitosis, then undergo fast and slow transition, respectively. This effect is dependent on the length of mitotic arrest and irrespective of cell fate or drug.

We demonstrate numerically that these rates increase with the duration of mitotic arrest within the 72-hour experimental time window. Additionally, given that the cellular fate is known, the hazard rates are identical among the different antimitotic drugs. This result is based on a previously unrecognized fact emerging from our quantitative analysis, *i.e*., that the proportions of RKO cells that survive until time “a” in mitotic arrest and subsequently undergo death in mitosis and mitotic slippage are identical when cells are exposed to nocodazole, AZ138, and monastrol, but significantly different for taxol. Moreover, we demonstrate that RKO cells display a higher hazard of undergoing death in mitosis than mitotic slippage throughout the 72-hour experimental time-course.

Our mathematical model is one of the first studies of its kind to provide the cellular apoptosis and slippage rates and their dependency on the length of mitotic arrest for the death in mitosis and mitotic slippage pathways in the RKO cell line. Overall, our results indicate that RKO cells exhibit a triphasic response curve irrespective of cell fate or antimitotic drug type or dose. We note that for each drug, the highest dose used was the smallest one required to block cell division, see Figs S4, 5 in^13^ Interestingly, our quantitative analysis suggests that the taxol-treated RKO cells display the slowest cell death in mitosis responses across all antimitotic drugs. Despite taxol being the slowest inducer of cell death in mitosis in the RKO cell line as evidenced by its specific hazard function, its slow induction of cell death is not a good measure for predicting its likelihood to induce death in mitosis, as 98% of RKO cells exposed to taxol do undergo death in mitosis following a prolonged mitotic arrest. Further investigations are needed to establish whether this is a concentration-dependent effect.

Based on our results, we formulate hypotheses on the dynamics behind the death in mitosis and mitotic slippage pathways in RKO cells. These have the potential to expand our understanding of the mechanisms which dictate whether a cell dies or survives a prolonged mitotic arrest, if tested in more focused experiments.

## Methods

### Data and modeling calibration

**Figure 1:**
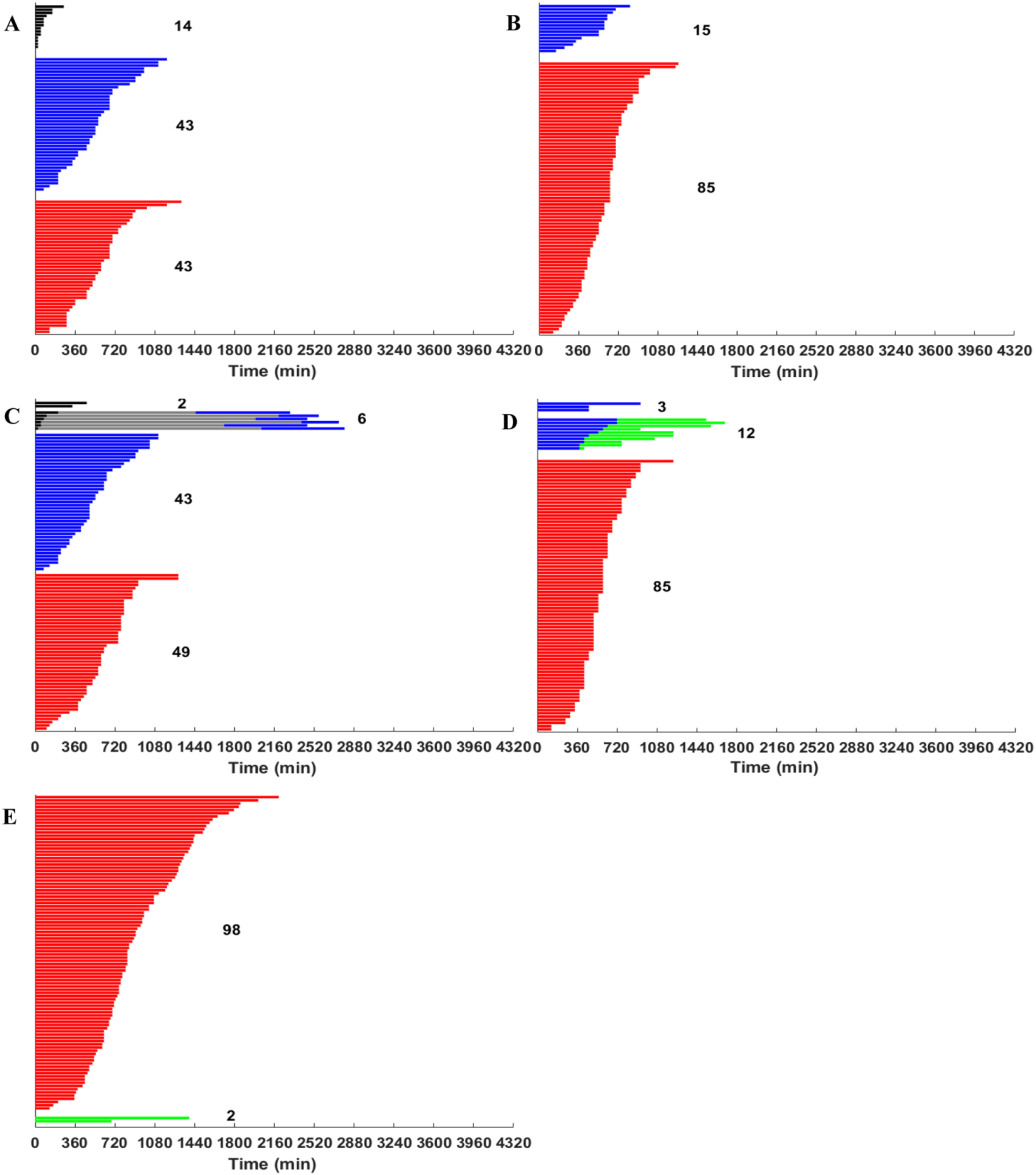
RKO cell response following prolonged exposure to antimitotic drugs during a 72-hour imaging period. RKO cell response to (**A**) 0.03 µM AZ138, (**B**) 1 µM AZ138, (**C**) 100 µM monastrol, (**D**) 30 ng/mL nocodazole, and (**E**) 0.1 µM taxol during a 72-hour imaging period. Data are adapted from the experimental findings reported in Fig. S5A in^13^. Each horizontal bar represents the fate of a single RKO cell. In response to the prolonged drug exposure, cells can either: (i) successfully divide (black bars), (ii) undergo mitotic slippage and remain in interphase throughout the duration of the experiment (blue bars), (iii) die in mitosis (red bars), (iv) undergo division, remain in interphase, then enter a second mitosis from which they slip and remain in interphase throughout the duration of the experiment (black, grey, and blue bars), (v) undergo mitotic slippage then die in interphase (blue and green bars); or (vi) die in interphase without having entered mitosis (green bars). For each panel (**A**–**E**), 100 distinct cell responses are represented. All reported values are in minutes. The number of cells corresponding to each category is shown in bold black.

In^13^ RKO cells in culture were continuously incubated with 0.03 µM AZ138, 1 µM AZ138, 100 µM monastrol, 30 ng/mL nocodazole, and 0.1 µM taxol during a 72-hour imaging period (Fig. 1A–E, respectively). We note that these drug concentrations represent equivalent, minimal saturating dosages of antimitotics required to ensure the efficient induction of mitotic arrest and a maximal induction of a 4 N DNA peak, as analyzed by flow cytometry and reported in Fig. S4B in^13^ The times spent in mitosis (Fig. S5A and C in^13^, red bars), or in mitosis following drug addition and before slippage (Fig. S5A and C in^13^, blue bars) were subsequently recorded. Therein, “0 min” on the x-axis of the cell fate profiles observed in Fig. S5A and C in^13^ represents the time when cells entered mitosis (K. Gascoigne, personal communication).

In^13^, data were pooled from recordings performed on individual cells synchronized in early S phase, using a thymidine block. Thymidine was added for 16 hours, before cells were released from the block. Drug medium was subsequently added 4.5 hours later. Imaging using automated time-lapse light microscopy was started at the same time. Images of RKO cells were then collected every 5 minutes for a total duration of 4320 minutes, equivalent to 72 hours (see Fig. 1A in^13^ for a timeline of the setup). Therein, mitosis was defined as the cellular state between nuclear envelope breakdown and the onset of anaphase (Fig. S1 in^13^). We note that the subsequent cell fates to antimitotic drugs were recorded either in the absence or presence of the pan-caspase inhibitor Boc-D-FMK, as represented in Fig. S5A in^13^ and Supplementary Fig. 1A–P in the current text.

In these experimental findings, fewer than 5% of the total number of RKO cells were reported to have successfully completed mitosis and divided into daughter cells in response to the microtubule-targeting agents nocodazole and taxol, and the spindle-targeting Eg5 inhibitors AZ138 and monastrol (see Fig. 1A–E for representative RKO cell responses). We note that in the absence of any antimitotic drugs, unsynchronized RKO cells are observed to undergo approximately three mitoses during a 72-hour imaging period^28^. Additionally, since in the experimental setup, the cells were spatially separated, the quantitative live-cell imagining technique employed by^13^ reported individual cell behavior, independent of spatial or global density considerations. As a result, we did not consider an explicit cellular density or a spatial component in our mathematical model. Subsequent results reported below are based on the data reported in Fig. 1A–E and Supplementary Fig. 1A–P. We note that we only considered the most predominant cell fate responses (*i.e*., ≥ 20 cells per cell fate).

### Statistical tests

We chose to focus on the predominant fates experienced by the RKO cells under prolonged antimitotic drug exposure, *i.e*., the fates governed by the death in mitosis and mitotic slippage pathways. To determine any statistically significant differences between the different RKO cell responses under prolonged exposure to the specific antimitotic drugs reported above, we first use the non-parametric Kruskal-Wallis test (or one-way ANOVA test for ranks) for n = 5 independent samples^38^. These samples correspond to the five RKO populations that undergo death in mitosis following exposure for a 72-hour period to 0.03 µM AZ138, 1 µM AZ138, 100 µM monastrol, 30 ng/mL nocodazole, and 0.01 µM taxol, respectively. We note that this test indicates whether the samples tested originate from the same distribution and identifies whether at least one of these samples is statistically significant. It does not, however, indicate in which sample(s) this dominance occurs.

We then performed pairwise non-parametric Mann-Whitney tests using all possible combinations between the groups of cells that died in mitosis (red bars) or that underwent mitotic slippage (blue bars); n.s., non-significant, *p < 0.01, **p < 0.001, ***p < 0.0001^38,39^. We note that this test is a nonparametric test that assesses whether two independent samples have similarly ranked distributions. It does not require the assumption of normal distributions. All statistical tests were two-tailed. The Holm-Bonferroni correction for multiple comparisons was used to calculate sequential corrected p-values, with α = 0.01 set as the determined significance threshold for rejecting the null hypothesis of samples having similarly ranked distributions. This procedure is used to control the familywise Type I error rate and reduce the risk of a Type II error as compared to using the simple Bonferroni correction.

### Distribution of times spent in mitotic arrest before dying or slipping out of mitosis

**Figure 2:**
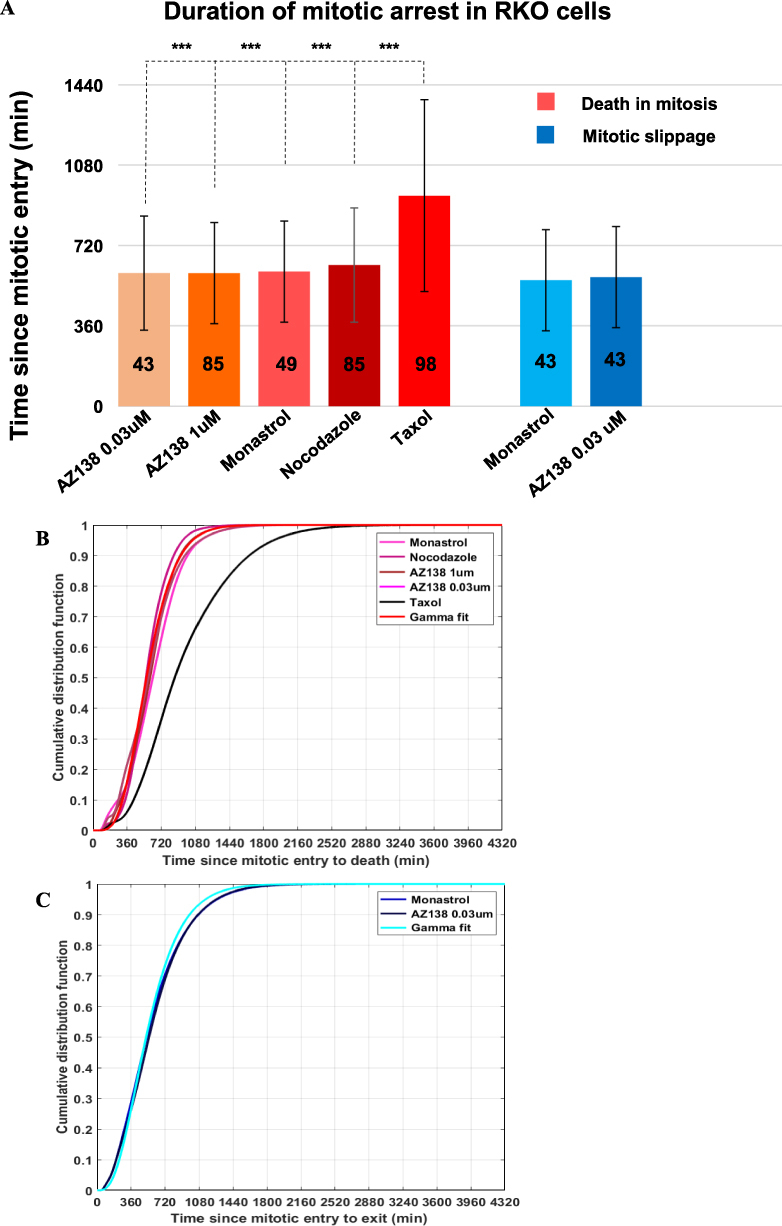
The time-dependent probability cells die in mitosis or slip is identical for all drugs except for taxol. (**A**) Statistical differences between the different RKO cell responses under prolonged exposure to a specific antimitotic drug were analyzed using the non-parametric Mann-Whitney test; n.s., non-significant, *p < 0.01, **p < 0.001, ***p < 0.0001. Pairwise comparisons were performed among all possible combinations between the groups of cells that died in mitosis (red bars) or that underwent mitotic slippage (blue bars). The vertical bar plots represents the mean ± s.d. duration of the drug-induced mitotic arrest in either death in mitosis (red bars) or mitotic slippage (blue bars). The reported values are in minutes. The number of cells corresponding to each category is shown in bold black inside each vertical bar plot. The cumulative distribution functions (CDF) for (**B**) death in mitosis and (**C**) mitotic slippage show the fraction of RKO cells that either died or slipped after entering mitosis as a function of time. Data are adapted from the experimental findings reported in Fig. S5A in^13^. Therein, the RKO cellular fate following prolonged exposure to four different drugs (monastrol, nocodazole, taxol and AZ138) was measured, based on the duration of drug-induced mitotic arrest. Cell death or slippage responses across drugs can be characterized by the cell-cycle age “a”-dependent Gamma distribution ${\rm{\Gamma }}({\rm{a}};\,{\rm{k}},\,{\rm{\theta }})$, with shape parameter k and scale parameter θ.

We use the experimental data to obtain the empirical cumulative density function (CDF) for the times spent in mitotic arrest corresponding to the death in mitosis and mitotic slippage pathways (illustrated in Fig. 2A–C). This is done by fitting a kernel smoothing function estimate to the CDF describing the duration of mitotic arrest corresponding to each drug, describing the duration of mitotic arrest reported in Fig. S5A,B in^13^. The procedure is performed using MATLAB’s “ksdensity” function. This function returns a cumulative density function, based on the sampled data. The amount of time a cell spends in mitotic arrest is thus assumed to be a continuous variable. The empirical CDF corresponding to each drug obtained using the kernel smoothing procedure is illustrated for cells that die in mitosis or slip out of mitosis in Figs. 2B or C, respectively.

### Polynomial fitting

To quantify the transition rates from mitotic arrest to cell death or interphase, we perform a polynomial least-squares fitting to the empirical transition rate derived from the Gamma CDF representing the fraction of RKO cells that either died or slipped after entering mitosis. The fitting procedure is done using MATLAB’s “polyfit”, “polyval” and “polyfix” functions^40^. The first function returns the coefficients for a polynomial of a user-specified degree, that represents the best fit in the least squares sense for the input data. The second is used to evaluate the fitted polynomials on a prescribed set of gridpoints. It also obtains error estimates in the Root-Mean-Square-Error (RMSE) sense between the approximate and fitted transitions rates from mitotic arrest to death in mitosis and mitotic slippage. The third function computes the coefficients for a polynomial of a user-specified degree, that represents the best fit in the least squares sense for the input data, with the added constraint that the polynomial must pass through a user-specified value at a specific point^40^. To best describe the corresponding transition rates from mitosis to death in mitosis for the non-taxol and taxol drugs, as well as the transition rate from mitosis to mitotic slippage, we chose to fit piecewise linear polynomials to the empirical data. Using linear polynomials, as opposed to higher-degree polynomials, enables us to easily interpret the modeling results into biologically meaningful observations that could be further tested with more focused experiments.

### Modeling approach

To study the emerging heterogeneity in RKO cell responses to prolonged antimitotic drug exposure, we model the dynamics of the RKO cancer cell population as the following system:

1 $$\frac{{\rm{d}}}{{\rm{d}}{\rm{a}}}{\bf{M}}({\rm{a}})=-\,(\begin{array}{cc}{\alpha }_{{\rm{M}}{\rm{A}}}({\rm{a}}) & 0\\ 0 & {\alpha }_{{\rm{M}}{\rm{I}}}({\rm{a}})\end{array}){\bf{M}}({\rm{a}})$$

with initial conditions $${\bf{M}}(0)=(\begin{array}{c}{\rm{p}}\\ 1-p\,\end{array}){{\rm{M}}}_{{\rm{t}}{\rm{o}}{\rm{t}}{\rm{a}}{\rm{l}}}.$$

Herein, the vector **M**(a) denotes the mitotic compartment, where the first component denotes the number of cells that are still in mitosis at time “a” and will undergo death in mitosis later on during the experiment. The second component of **M**(a) denotes the number of cells that are still in mitosis at time “a” and will slip out of mitosis into interphase later on during the experiment. The rate of change of **M**(a) with respect to the experimental time course “a” (*i.e*., cell-cycle age) is represented by $\frac{{\rm{d}}}{{\rm{da}}}.$ The derivative $\frac{{\rm{d}}}{{\rm{da}}}{\bf{M}}({\rm{a}})$ implies that mitotic cells advance in cell-cycle age as time progresses.

From mitotic arrest, cells transition with time-dependent rate ${{\rm{\alpha }}}_{{\rm{MA}}}({\rm{a}})$ and probability p to intrinsic cell death (*i.e*., apoptosis) or slip out of mitosis into interphase with time-dependent rate α_MI_(a) and probability 1−p. In doing so, we implicitly assume that the death in mitosis and mitotic slippage pathways are simultaneously active, but mechanistically independent of each other during mitotic arrest. This assumption is supported experimentally by various cancer cell studies^3,13,14,18,26,27^, and numerically by predictive modeling in^18^.

The total number of RKO cells exposed to 0.03 µM AZ138, 1 µM AZ138, monastrol, nocodazole, and taxol that undergo either death in mitosis or mitotic slippage, as depicted in Fig. 1A–E, is:

$${{\rm{M}}}_{{\rm{t}}{\rm{o}}{\rm{t}}{\rm{a}}{\rm{l}}}=\{\begin{array}{c}\begin{array}{c}\,86\,\\ 85\\ 92\\ 85\\ 98\end{array}\end{array}{\rm{c}}{\rm{e}}{\rm{l}}{\rm{l}}{\rm{s}},\,{\rm{r}}{\rm{e}}{\rm{s}}{\rm{p}}{\rm{e}}{\rm{c}}{\rm{t}}{\rm{i}}{\rm{v}}{\rm{e}}{\rm{l}}{\rm{y}}.$$

Thus, the initial number of RKO cells arrested in mitosis, corresponding to each drug and cell fate, as evidenced in Fig. 1A–E is: $${\bf{M}}{(0)}_{0.03{\rm{\mu }}\text{M }{\rm{A}}{\rm{Z}}138}=\,(\begin{array}{c}43\\ 43\end{array}),$$ $${\bf{M}}{(0)}_{\text{1}{\rm{\mu }}\text{M }{\rm{A}}{\rm{Z}}138}=(\begin{array}{c}85\\ 0\end{array})$$, $${\bf{M}}{(0)}_{{\rm{m}}{\rm{o}}{\rm{n}}{\rm{a}}{\rm{s}}{\rm{t}}{\rm{r}}{\rm{o}}{\rm{l}}}=(\begin{array}{c}49\\ 43\end{array}),$$ $${\bf{M}}{(0)}_{{\rm{n}}{\rm{o}}{\rm{c}}{\rm{o}}{\rm{d}}{\rm{a}}{\rm{z}}{\rm{o}}{\rm{l}}{\rm{e}}}=(\begin{array}{c}85\\ 0\end{array})$$ $${\rm{a}}{\rm{n}}{\rm{d}}\,{\bf{M}}{(0)}_{{\rm{t}}{\rm{a}}{\rm{x}}{\rm{o}}{\rm{l}}}=(\begin{array}{c}98\\ 0\end{array}),$$ which yields a drug type- and dose-dependent probability p of undergoing death in mitosis following mitotic arrest of $\frac{1}{2},\,1,\,\frac{49}{92},\,1,\,{\rm{and}}\,1$in the case of 0.03 µM AZ138, 1 µM AZ138, monastrol, nocodazole, and taxol, respectively.

The solution of the linear system in Equation (1) is:

2 $${\bf{M}}({\rm{a}})=(\begin{array}{c}{{\rm{p}}{\rm{e}}}^{-{\int }_{0}^{{\rm{a}}}{\alpha }_{{\rm{M}}{\rm{A}}}({{\rm{a}}}^{\text{'}}){{\rm{d}}{\rm{a}}}^{\text{'}}}\\ (1-{\rm{p}}){{\rm{e}}}^{-{\int }_{0}^{{\rm{a}}}{\alpha }_{{\rm{M}}{\rm{I}}}({{\rm{a}}}^{\text{'}}){{\rm{d}}{\rm{a}}}^{\text{'}}}\end{array}){{\rm{M}}}_{{\rm{t}}{\rm{o}}{\rm{t}}{\rm{a}}{\rm{l}}}.$$

In order to determine the hazard functions corresponding to the RKO cells undergoing death in mitosis and slippage, we estimate the proportion of RKO cells that survive until time “a” in mitotic arrest and subsequently undergo death in mitosis or mitotic slippage as the exponentially decaying process:

3 $$(\begin{array}{c}{{\rm{e}}}^{-{\int }_{0}^{{\rm{a}}}{\alpha }_{{\rm{M}}{\rm{A}}}({\rm{a}}^{\prime} )d{\rm{a}}^{\prime} }\\ {{\rm{e}}}^{-{\int }_{0}^{{\rm{a}}}{\alpha }_{{\rm{M}}{\rm{I}}}({\rm{a}}^{\prime} )d{\rm{a}}^{\prime} }\end{array})\sim \overline{{\bf{F}}({\bf{a}})}=(\begin{array}{c}1-{{\rm{F}}}_{{\rm{M}}{\rm{A}}}({\rm{a}};\,{\rm{k}},\,\theta )\\ 1-{{\rm{F}}}_{{\rm{M}}{\rm{I}}}({\rm{a}};\,{\rm{k}},\,\theta )\end{array})$$

where the cell death or slippage responses across drugs are characterized by the cell-cycle age “a”-dependent Gamma CDF ${{\rm{F}}}_{{\rm{MA}}}({\rm{a}};\,{\rm{k}},\,{\rm{\theta }})$ or ${{\rm{F}}}_{{\rm{MI}}}({\rm{a}};\,{\rm{k}},\,{\rm{\theta }})$, corresponding to the death in mitosis and mitotic slippage pathways, respectively. Here, the notation “~” represents “is distributed as”.

Each Gamma CDF models the fraction of RKO cells that either die or slip after entering mitosis as a function of time, and is characterized by its corresponding shape kand scale parameters θ (see Fig. 2B,C for the quantification). Herein, $\overline{{\bf{F}}({\bf{a}})}$ denotes the vector of survival functions corresponding to each pathway, where the survival function is defined as 1-CDF.

To determine ${{\rm{\alpha }}}_{{\rm{MA}}}({\rm{a}})$ and ${{\rm{\alpha }}}_{{\rm{MI}}}({\rm{a}})$, we obtain from Equation (3):

4 $$(\begin{array}{c}{\int }_{0}^{{\rm{a}}}{\alpha }_{{\rm{M}}{\rm{A}}}({\rm{a}}^{\prime} ){\rm{d}}{\rm{a}}^{\prime} \\ {\int }_{0}^{{\rm{a}}}{\alpha }_{{\rm{M}}{\rm{I}}}({\rm{a}}^{\prime} ){\rm{d}}{\rm{a}}^{\prime} \end{array})\approx (\begin{array}{c}-\,{\rm{l}}{\rm{o}}{\rm{g}}[1-{{\rm{F}}}_{{\rm{M}}{\rm{A}}}({\rm{a}};\,{\rm{k}},\,\theta )]\\ -\,{\rm{l}}{\rm{o}}{\rm{g}}[1-{{\rm{F}}}_{{\rm{M}}{\rm{I}}}({\rm{a}};\,{\rm{k}},\,\theta )]\end{array}).\,$$

Taking the discrete derivative of Equation (4) yields:

5 $$(\begin{array}{c}\frac{{\int }_{0}^{{\rm{a}}+{\rm{\Delta }}{\rm{a}}}{\alpha }_{{\rm{M}}{\rm{A}}}({\rm{a}}^{\prime} ){\rm{d}}{\rm{a}}^{\prime} \,-\,{\int }_{0}^{{\rm{a}}}{\alpha }_{{\rm{M}}{\rm{A}}}({\rm{a}}^{\prime} ){\rm{d}}{\rm{a}}^{\prime} }{{\rm{\Delta }}a}\\ \frac{{\int }_{0}^{{\rm{a}}+{\rm{\Delta }}{\rm{a}}}{\alpha }_{{\rm{M}}{\rm{I}}}({\rm{a}}^{\prime} ){\rm{d}}{\rm{a}}^{\prime} \,-\,{\int }_{0}^{{\rm{a}}}{\alpha }_{{\rm{M}}{\rm{I}}}({\rm{a}}^{\prime} ){\rm{d}}{\rm{a}}^{\prime} \,}{{\rm{\Delta }}a}\end{array})\approx (\begin{array}{c}{\alpha }_{{\rm{M}}{\rm{A}}}({\rm{a}})\\ {\alpha }_{{\rm{M}}{\rm{I}}}({\rm{a}})\end{array})\approx (\begin{array}{c}\frac{{{\rm{f}}}_{{\rm{M}}{\rm{A}}}({\rm{a}};\,{\rm{k}},\,\theta )}{1-{{\rm{F}}}_{{\rm{M}}{\rm{A}}}({\rm{a}};\,{\rm{k}},\,\theta )}\\ \frac{{{\rm{f}}}_{{\rm{M}}{\rm{I}}}({\rm{a}};\,{\rm{k}},\,\theta )}{1-{{\rm{F}}}_{{\rm{M}}{\rm{I}}}({\rm{a}};\,{\rm{k}},\,\theta )}\end{array})$$

where “${\rm{\Delta }}a$” represents the discrete time-step, which is set in our numerical simulations to one minute.

We note that the right-hand side of Equation (5) is equal to $$(\begin{array}{c}-\frac{{\rm{d}}}{{\rm{d}}{\rm{a}}}\,{\rm{l}}{\rm{o}}{\rm{g}}[1-{{\rm{F}}}_{{\rm{M}}{\rm{A}}}({\rm{a}};\,{\rm{k}},\,\theta )]\\ -\frac{{\rm{d}}}{{\rm{d}}{\rm{a}}}\,{\rm{l}}{\rm{o}}{\rm{g}}[1-{{\rm{F}}}_{{\rm{M}}{\rm{I}}}({\rm{a}};\,{\rm{k}},\,\theta )]\end{array})$$, with ${{\rm{f}}}_{{\rm{MA}}}({\rm{a}};\,{\rm{k}},\,{\rm{\theta }})$ and ${{\rm{f}}}_{{\rm{MI}}}({\rm{a}};\,{\rm{k}},\,{\rm{\theta }})$ representing the Gamma probability distribution functions corresponding to the fraction of RKO cells that either died or slipped after entering mitosis as a function of time “a”, respectively.

### Availability of Materials and Data

All data generated or analyzed during this study are included in this published article.

## Results

### The time-dependent probability cells die in mitosis or slip is identical for all drugs except for taxol

To determine whether the type of antimitotic drug used affects the duration of mitotic arrest in RKO cells, we compare the variable durations of mitotic arrest illustrated in Fig. 1A–E corresponding to cells that either died in mitosis or exited mitosis and returned to interphase. We first analyze the statistical differences between the different RKO cell responses under prolonged exposure to a specific antimitotic drug using the non-parametric Kruskal-Wallis test for n = 5 independent samples. These correspond to the five RKO populations that undergo death in mitosis following exposure for a 72-hour period to 0.03 µM AZ138, 1 µM AZ138, 100 µM monastrol, 30 ng/mL nocodazole, and 0.01 µM taxol, respectively. The observed aggregate difference among the five samples was significant beyond the <0.0001 significance level (data not shown).

We then performed pairwise non-parametric Mann-Whitney tests using all possible combinations between the groups of cells that died in mitosis (red bars) or that underwent mitotic slippage (blue bars); n.s., non-significant, *p < 0.01, **p < 0.001, ***p < 0.0001. RKO cells exposed to taxol exhibit a markedly distinct response to the prolonged taxol exposure, *i.e*., the duration of mitotic arrest induced by taxol in RKO cells is significantly different compared to the durations of the arrest induced by nocodazole, monastrol, or AZ138. This effect achieves statistical significance beyond the < 0.001 level (Fig. 2A). This effect is however, not conserved when RKO cells are exposed to taxol in the presence of the pan-caspase inhibitor (see Supplementary Fig. 2A).

### Cell death and slippage responses across drugs can be characterized by Gamma distributions

As indicated by our statistical analysis, the time-dependent probability cells undergo death in mitosis or mitotic slippage is identical for all drugs except for taxol. To best describe the duration of mitotic arrest cells experience before dying in mitosis or slipping from mitosis and returning to interphase, we chose to represent the RKO cell death or slippage responses across drugs by corresponding Gamma distributions ${\rm{\Gamma }}({\rm{a}};\,{\rm{k}},\,{\rm{\theta }})$. Each distribution represents the fraction of RKO cells that either died or slipped after entering mitosis as a function of time (*i.e*., cell-cycle age “a”), is characterized by its corresponding shape k and scale parameters θ, as illustrated in Fig. 2B,C. The choice of the Gamma distribution to model the duration of mitotic arrest is motivated by this distribution’s asymmetry and right-skewness, and is confirmed by the excellent fit to the empirical data.

Specifically, in Fig. 2B the death in mitosis CDF for the non-taxol drugs can be represented by ${\rm{\Gamma }}(5.91,\,101.15),$with 95% confidence intervals [5, 6.98] and [85, 120.36] for k and θ, respectively. The RMSE between the empirical CDF (*i.e*., based on the sampled data and obtained by using MATLAB’s “ksdensity” function) and the Gamma fit is equal to $1.1\cdot {10}^{-2}$. Similarly, the death in mitosis CDF for taxol can be represented by ${\rm{\Gamma }}(4.43,\,212.9),$ with 95% confidence intervals [3.38, 5.8] and [159.9, 283.5] for k and θ, respectively. The RMSE between the empirical CDF and the Gamma fit is equal to $1.23\cdot {10}^{-2}$. In Fig. 2C, the mitotic slippage CDF can be represented by ${\rm{\Gamma }}(3.55,\,161),$ with 95% confidence intervals [2.66, 4.72] and [118.4, 218.8] for k and θ, respectively. The RMSE between the empirical CDF and the Gamma fit is equal to $1.2\cdot {10}^{-2}$.

We note that using the Gamma CDF instead of the empirical CDF obtained by using MATLAB’s “ksdensity” function enables us in subsequent simulations to obtain a closed-form expression for the age-dependent transition rates from mitosis to death in mitosis and mitotic slippage, *i.e*., ${{\rm{\alpha }}}_{{\rm{MA}}}({\rm{a}})$ and ${{\rm{\alpha }}}_{{\rm{MI}}}({\rm{a}})$, respectively, as demonstrated in Equations (3–5).

Interestingly, when investigating HCT116 and HT29 colon carcinoma cell line responses to the antimitotic drugs, cell death and slippage responses can also be well characterized by Gamma distributions. For each corresponding cell fate, a distinct Gamma CDF fits the cell profiles independent of the antimitotic drug used, see Supplementary Figs 4A–I and 5A–I for the HT29 and HCT116 cell line responses, respectively. This effect is conserved in the presence of the pan-caspase inhibitor for all three cell lines under study, see Supplementary Figs 3–5.

### The mitotic arrest time does not dictate cell fate

**Figure 3:**
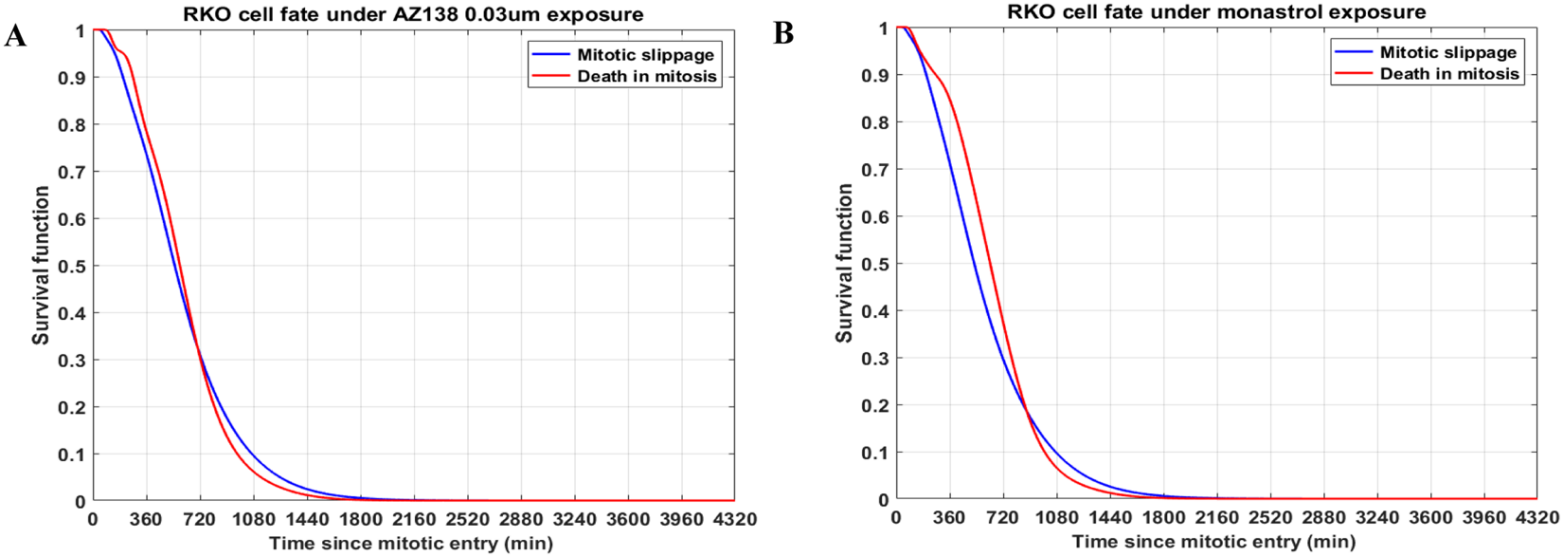
For shorter durations of mitotic arrest, RKO cells are slightly more likely to slip in interphase, while for longer durations of mitotic arrest, RKO cells are slightly more likely to die in mitosis. Under prolonged (**A**) 0.03 µM AZ138 and (**B**) 100 µM monastrol exposure, RKO cells are more likely to undergo mitotic slippage (blue lines) rather than death in mitosis (red lines) for a shorter duration of mitotic arrest, *i.e*., 11.73 and 14.65 hours, respectively.

Under prolonged 0.03 µM AZ138 and 100 µM monastrol exposure, RKO cells are slightly more likely to undergo mitotic slippage (blue lines in Fig. 3A,B) rather than death in mitosis (red lines in Fig. 3A,B) for a shorter duration of mitotic arrest, *i.e*., 11.73 and 14.65 hours, respectively. However, for durations longer than 11.73 and 14.65 hours of mitotic arrest in RKO cells exposed to 0.03 µM AZ138 and 100 µM monastrol, respectively, cells are slightly more likely to undergo death in mitosis rather than mitotic slippage. We additionally report the RKO cell fate in the death in mitosis and mitotic slippage pathways under nocodazole and taxol prolonged exposure in the presence of the pan-caspase inhibitor Boc-D-FMK in Supplementary Fig. 3F,G. We also report HCT116 cell fate in the death in mitosis and mitotic slippage pathways under monastrol, nocodazole, AZ138 µM and taxol in the presence and absence of the pan-caspase inhibitor in Supplementary Fig. 1J–N. We did not observe any significant differences in the CDFs corresponding to the death in mitosis and mitotic slippage pathways.

### RKO cells exhibit a triphasic response curve irrespective of cell fate or antimitotic drug

**Figure 4:**
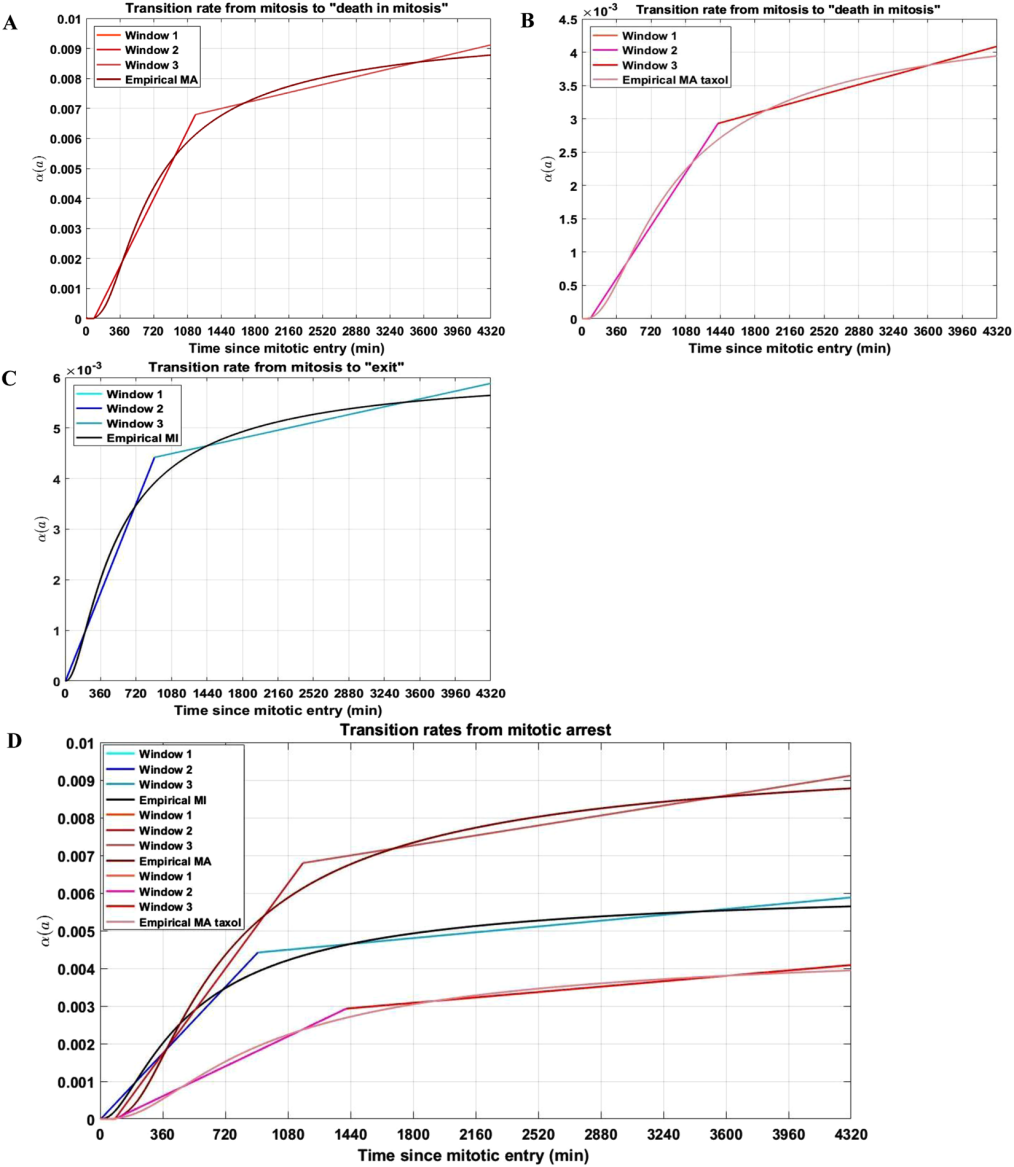
The hazard functions corresponding to the RKO cells undergoing death in mitosis and slippage increase with prolonged mitotic arrest, and exhibit a triphasic response irrespective of cell fate or antimitotic drug used. The hazard functions corresponding to the RKO cells undergoing death in mitosis for (**A**) non-taxol drugs and (**B**) taxol and to the RKO cells undergoing mitotic slippage (**C**) increase monotonically with time, *i.e*., the duration of mitotic arrest. Each labeled “Window” in the figure legends corresponds to time period during the mitotic arrest RKO cells undergo one of the following: (i) remain in mitotic arrest with probability 1 (“Window 1” in Fig. 4A–C legend), (ii) fast transition from mitotic arrest to cell death in mitosis, or mitotic slippage (“Window 2” in Fig. 4A–C legend, respectively), or (iii) slow transition from mitotic arrest to cell death in mitosis, or mitotic slippage (“Window 3” in Fig. 4A–C legend, respectively). The hazard functions ${{\rm{\alpha }}}_{{\rm{MA}}}({\rm{a}})$ and ${{\rm{\alpha }}}_{{\rm{MI}}}({\rm{a}})$, corresponding to the death in mitosis induced by non-taxol drugs, taxol (red lines) and mitotic slippage (blue lines), derived empirically from solving Equation (5) are illustrated as the non-linear functions in Fig. 4A–C, respectively. (**D**) To better visualize and compare RKO cell fate responses across drugs, the distinct hazard functions corresponding to the death in mitosis responses induced by the non-taxol drug, taxol, and mitotic slippage responses are plotted.

From mitotic arrest, cells undergo death in mitosis (*i.e*., apoptosis) with probability p and age-dependent transition rate ${{\rm{\alpha }}}_{{\rm{MA}}}({\rm{a}})$. Alternatively, they can undergo mitotic slippage and return to interphase with probability 1−p and age-dependent transition rate ${{\rm{\alpha }}}_{{\rm{MI}}}({\rm{a}})$, as shown in Equation (1). We note that these functions increase with prolonged mitotic arrest, irrespective of cell fate or antimitotic drug, see Fig. 4A–D.

We subsequently fit piecewise linear polynomials to the transition rates from mitosis to apoptosis,${{\rm{\alpha }}}_{{\rm{MA}}}({\rm{a}}),$ for the non-taxol and taxol drugs, as illustrated in Fig. 4A,B, respectively (red bars), and from mitosis to slippage into interphase, as illustrated in Fig. 4C (blue bars). Each labeled “Window” in the Fig. 4 legends corresponds to time period during the mitotic arrest RKO cells undergo one of the following: (i) remain in mitotic arrest (“Window 1” in Fig. 4A–D legend), (ii) fast transition from mitotic arrest to cell death in mitosis, or mitotic slippage (“Window 2” in Fig. 4A–D legend, respectively), or (iii) slow transition from mitotic arrest to cell death in mitosis, or mitotic slippage (“Window 3” in Fig. 4A–D legend, respectively). We note that the fast and slow transitions refer to the slope of fitted linear polynomials corresponding to “Window 2” and “Window 3”, respectively. The slopes corresponding to “Window 2” are bigger than the ones corresponding to “Window 3” throughout Fig. 4A–D.

The piecewise linear polynomials that best describe the hazard functions corresponding to the death in mitosis and mitotic slippage cell responses are reported in Table 1. The RMSE values between the empirically-derived and fitted ${{\rm{\alpha }}}_{{\rm{MA}}}({\rm{a}})$ and ${{\rm{\alpha }}}_{{\rm{MI}}}({\rm{a}})$ corresponding to the death in mitosis and mitotic slippage, respectively, can also be found in Table 1. Overall, RKO cells display a higher hazard of undergoing death in mitosis than mitotic slippage throughout the 72-hour experimental time-course (top red and blue lines in Fig. 4D). Moreover, the transition from mitotic arrest to cell death in mitosis for the non-taxol drugs (“Windows 2–3” in Fig. 4A) is overall faster than the transition from mitotic arrest to mitotic slippage (“Windows 2–3” in Fig. 4C), with a 1.3–1.7-fold difference in the slopes of the piecewise linear polynomials corresponding to the two alternative pathways.

**Table 1:** The piecewise linear polynomials that best describe the hazard functions corresponding to the death in mitosis cell responses to the non-taxol and taxol drugs, and to the mitotic slippage cell responses.

Type of cell response	Piecewise linear polynomial	RMSE between empirical and fitted polynomial	Corresponding figure
Death in mitosis induced by non-taxol drugs	$${\alpha }_{{\rm{M}}{\rm{A}}}({\rm{a}})=\{\begin{array}{c}\begin{array}{c}0,\,{\rm{f}}{\rm{o}}{\rm{r}}\,0^{\prime} \le a\le 82^{\prime} ,\\ 6.28\cdot {10}^{-6}a-5.34\cdot {10}^{-4},\,{\rm{f}}{\rm{o}}{\rm{r}}\,82^{\prime} \le a\le 1164^{\prime} ,\\ 7.36\cdot {10}^{-7}a+5.9\cdot {10}^{-3},\,{\rm{f}}{\rm{o}}{\rm{r}}\,1164^{\prime} \le a\le 4320^{\prime} .\end{array}\end{array}$$	$3.27\cdot {10}^{-4}$	4A
Death in mitosis induced by taxol	$${\alpha }_{{\rm{M}}{\rm{A}}}({\rm{a}})=\{\begin{array}{c}\begin{array}{c}0,\,{\rm{f}}{\rm{o}}{\rm{r}}\,\,0^{\prime} \le a\le 86^{\prime} ,\\ 2.2\cdot {10}^{-6}a-1.89\cdot {10}^{-4},\,{\rm{f}}{\rm{o}}{\rm{r}}\,86^{\prime} \le a\le 1416^{\prime} ,\\ 3.99\cdot \,{10}^{-7}a+2.4\cdot {10}^{-3},\,{\rm{f}}{\rm{o}}{\rm{r}}\,1416^{\prime} \le a\le 4320^{\prime} .\end{array}\end{array}$$	$1.25\cdot {10}^{-4}$	4B
Mitotic slippage	$${\alpha }_{{\rm{M}}{\rm{I}}}({\rm{a}})=\{\begin{array}{c}\begin{array}{c}0,\,{\rm{f}}{\rm{o}}{\rm{r}}\,0^{\prime} \le a\le 4^{\prime} ,\\ 4.89\cdot {10}^{-6}a-1.96\cdot {10}^{-5},\,{\rm{f}}{\rm{o}}{\rm{r}}\,4^{\prime} \le a\le 907^{\prime} ,\\ 4.29\cdot {10}^{-7}a+4\cdot {10}^{-3},\,{\rm{f}}{\rm{o}}{\rm{r}}\,907^{\prime} \le a\le 4320^{\prime} .\end{array}\end{array}$$	$2.34\cdot {10}^{-4}$	4C

We additionally note that the taxol-treated RKO cells display the slowest cell death in mitosis responses across all antimitotic drugs (Fig. 4B), as evidenced by 1.8–2.8-fold difference in the slopes of the piecewise linear polynomials corresponding to the non-taxol drugs and taxol, illustrated in Fig. 4D. Interestingly, despite taxol being the slowest inducer of cell death in mitosis in the RKO cell line as evidenced by its specific hazard function (illustrated in Fig. 4D), this observation is not a good measure for predicting its likelihood to induce death in mitosis, as 98% of RKO cells exposed to taxol do undergo death in mitosis following a prolonged mitotic arrest (see Fig. 1E herein and Fig. S5A in^13^). However, this effect might be dose-dependent, as in^13^, RKO cells were only exposed to 0.1 µM taxol during a 72-hour imaging period.

## Discussion

The mechanisms behind drug-induced prolonged mitotic arrest and cancer cell death using different antimitotic drugs have only recently begun to be elucidated using live quantitative cell imaging^13,14,19,20,32^. Using live quantitative single cell imaging, several studies have demonstrated that individual cancer cells display widely varying responses to antimitotic drugs. These studies have expanded our understanding of the mechanisms which determine whether a cell dies in mitosis or survives a prolonged mitotic arrest by returning to interphase following exposure to antimitotics.

For example, in^13^, the authors proposed a model where the two predominant cancer cell fates, *i.e*., mitotic slippage and death in mitosis, are governed by two independent networks. The first network involves the cell-cycle regulator cyclin B1 and its kinase partner Cdk1 as follows: an active anaphase promoting complex APC/C, an E3 ubiquitin ligase, targets cyclin B1 for proteasome degradation past the threshold necessary to maintain sufficient Cdk1 activity and promotes mitotic exit. Cells thus escape mitotic arrest without completing mitosis, which can lead to tetraploidy, senescence, or apoptosis following a subsequent mitosis^21,41,42^. The second network involves caspase activation and signal accumulation during mitotic arrest, the destabilization of the survivin/XIAP complex, and alterations in the intracellular localization and activation status of Bcl-2 family members^9,36^.

Several major questions regarding cancer cell fate and cell response to prolonged antimitotic therapies remain unresolved: (1) Does duration of mitotic arrest affect cell fate? (2) What are the cellular apoptosis and slippage rates corresponding to the death in mitosis and mitotic slippage? (3) Are these rates dependent on the length of mitotic arrest? and finally (4) Do any universal features in the cellular responses to the different antimitotics characterizing each pathway exist?^13,25,26,28,29,33^.

In this paper, we provide the cellular apoptosis and slippage rates and their dependency on the length of mitotic arrest for the death in mitosis and mitotic slippage pathways in the RKO cell line. We demonstrate numerically that these rates increase with the duration of mitotic arrest. Given the cellular fate is known, they are identical among the distinct non-taxol antimitotic drugs whose effect on RKO cell fate was investigated in^13^. Importantly, this is a previously unrecognized fact which emerges from our quantitative analysis, *i.e*., that the proportions of RKO cells that survive until time “a” in mitotic arrest and subsequently undergo death in mitosis and mitotic slippage are identical when cells are exposed to non-taxol drugs. Moreover, we demonstrate that RKO cells display a higher hazard of undergoing death in mitosis than mitotic slippage throughout the 72-hour experimental time-course. Additionally, our results indicate that RKO cells exhibit a triphasic response curve irrespective of cell fate or antimitotic drug. Interestingly, taxol induces the slowest cell death in mitosis responses across all antimitotic drugs in RKO cells. However, its slow induction of cell death is not a good measure for predicting its likelihood to induce death in mitosis, as experimentally, almost all RKO cells exposed to taxol do undergo death in mitosis following a prolonged mitotic arrest, as reported in^13,14^.

We now briefly comment upon several aspect emerging from our quantitative modeling results. First, it is intriguing that RKO cells exposed to the microtubule-destabilizing nocodazole, and Eg5-kinesin inhibitors AZ138 and monastrol exhibit triphasic responses to prolonged antimitotic exposure. To the best of our knowledge, this is a previously unrecognized fact. Our statistical analysis indicates that the duration of mitotic arrest induced by these drugs is not statistically different between these drugs, as both cells that die in mitosis or exit mitosis and slip into interphase display the same CDFs, respectively. While death in mitosis and slippage kinetics are highly variable from cell to cell, our results suggests that the microtubule-destabilizing nocodazole and Eg5-kinesin inhibitors AZ138 and monastrol induce the same duration of mitotic arrest in RKO cells corresponding to each pathway, despite the different drug targets and pharmacokinetics. This highlights a potential functional convergence between the different non-taxol antimitotic drugs used in the study with respect to inducing similar distributions of times spent in mitotic arrest before dying or slipping out of mitosis. This intriguing observation merits further experimental investigation. Furthermore, this effect might be dose-independent, as already observed with two different doses of AZ138 where cell death or slippage responses across different doses of the same drug could be characterized by a single Gamma distribution. Further investigations of such dose-response effects are warranted.

Second, RKO cells exposed to taxol exhibit a markedly distinct response to the prolonged taxol exposure, *i.e*., the duration of mitotic arrest induced by taxol in RKO cells is significantly longer compared to the durations of the arrest induced by nocodazole, monastrol, or AZ138. This effect achieves statistical significance beyond the < 0.001 level. Our results suggest that taxol is more efficient at inducing RKO cell death compared to the kinesin-5 inhibitors and nocodazole, but requires a longer duration of mitotic arrest to induce its proapoptotic effect compared to other antimitotic drugs, an observation also pointed out in^14^. The addition of the pan-caspase inhibitor to the taxol-treated cells rescues some RKO cells from death in mitosis, however we did not observe a statistically significant change between the death in mitosis CDFs corresponding to the taxol-treated cells in the presence or absence of the caspase inhibitor.

Third, our results indicate that the fraction of RKO cells that either die or slip after entering mitosis following continued exposure to nocodazole, monastrol, AZ138, and taxol can be well-approximated by Gamma distributions. Specifically, our results indicate that the shape parameter k of the Gamma distributions corresponding to the fraction of RKO cells that die in mitosis under non-taxol and taxol exposure, or slip back into interphase is 5.91, 4.43, or 3.95, respectively. Interestingly, this suggests the existence of a sequence of approximately six or four independent, exponentially distributed random variables, each modeling an event responsible for inducing RKO cell death by the non-taxol drugs, and taxol, respectively - see^43^ for a theoretical proof. In the case of the death in mitosis pathway, such a sequence could involve in chronological order events such as the activation of executive caspases, Bcl-xL expression levels, the depletion of the anti-apoptotic protein Mcl-1, increased microtubule stabilization leading to interference with cellular trafficking and microtubule-mediated cellular transport, and sequestration of Bax/Bak sufficient to trigger Mitochondrial Outer Membrane Permeabilization (MOMP). In the case of the mitotic slippage pathway, our results also suggest the existence of four independent exponentially distributed random variables responsible for inducing mitotic slippage and survival of the RKO cells in interphase. These events could, for example, be correlated with cyclin B1 level degradation, or the prolonged activation of Cdk1. Interestingly, when analyzing the HCT116 and HT29 colon carcinoma data in comparison to the RKO data, the shape parameters corresponding to the Gamma fits for the HT29 data were bigger than the ones corresponding to the Gamma fits for the RKO or HCT116 data (double-digit versus single-digit numbers). This suggests that the death in mitosis and mitotic slippage pathways for the HT29 cells could be governed by a bigger number of molecular events compared to the RKO or HCT116 cells.

Fourth, our results indicate that RKO cells exhibit a triphasic response curve irrespective of cell fate or antimitotic drug. To the best of our knowledge, this observation was previously unrecognized. Interestingly, RKO cells remain in mitotic arrest for periods of time shorter than 1.5 hours, then undergo a fast transition from arrest to death in mitosis or to mitotic slippage and return in interphase as long as the duration of mitotic arrest is shorter than 24 hours. If cells continue to remain in mitotic arrest for more than 24 hours, their subsequent transition to cellular death or slippage is slower compared to the first phase of transition.

In the current work, we provide an *in silico* modeling framework for studying the emerging heterogeneity in the response of the colon carcinoma RKO cell line to antimitotic drugs. Our *in silico* quantitative approach incorporates experimental results and uses mathematical models in order to better inform *in vitro* phenomena. Our modeling framework will serve as a basis for future studies of cancer cell heterogeneity *in vitro* of more complex responses in the presence of antimitotic drugs of both apoptosis-resistant and apoptosis-sensitive cell lines other than the colon carcinoma RKO, HCT116 and HT29 cell lines.

## Electronic supplementary material

Supplementary Information
