# Supplementary material for: Universal response in the RKO colon cancer cell line to distinct antimitotic therapies
Source: Sci Rep. 2018 Jun 12;8:8979. doi: 10.1038/s41598-018-27267-7 (PMC5997697; doi:10.1038/s41598-018-27267-7)
Supplement: Supplementary file 1 — Supplementary Information [file 41598_2018_27267_MOESM1_ESM.pdf]

Universal response in the RKO colon cancer cell line to distinct antimitotic therapies

Authors: Alexander Lorz[1, 2], Dana-Adriana Botesteanu[3, 4*], Doron Levy[4]**

Affiliations:

1 Computer, Electrical and Mathematical Sciences and Engineering Division, King Abdullah University of Science and Technology, Thuwal, Saudi Arabia

2 Sorbonne Universités, Université Pierre et Marie Curie Université Paris 06, Unité mixte de recherche 7598, Laboratoire Jacques-Louis Lions, Paris, France

3 Women's Malignancies Branch, Center for Cancer Research, National Cancer Institute, National Institutes of Health, Bethesda, Maryland, United States of America

4 Department of Mathematics and Center for Scientific Computation and Mathematical Modeling, University of Maryland, College Park, Maryland, United States of America

*Present address : Department of Discovery ADME, Boehringer Ingelheim RCV GmbH & Co KG, Vienna, Austria

****Corresponding author**

E-mail: dlevy@math.umd.edu (DL)

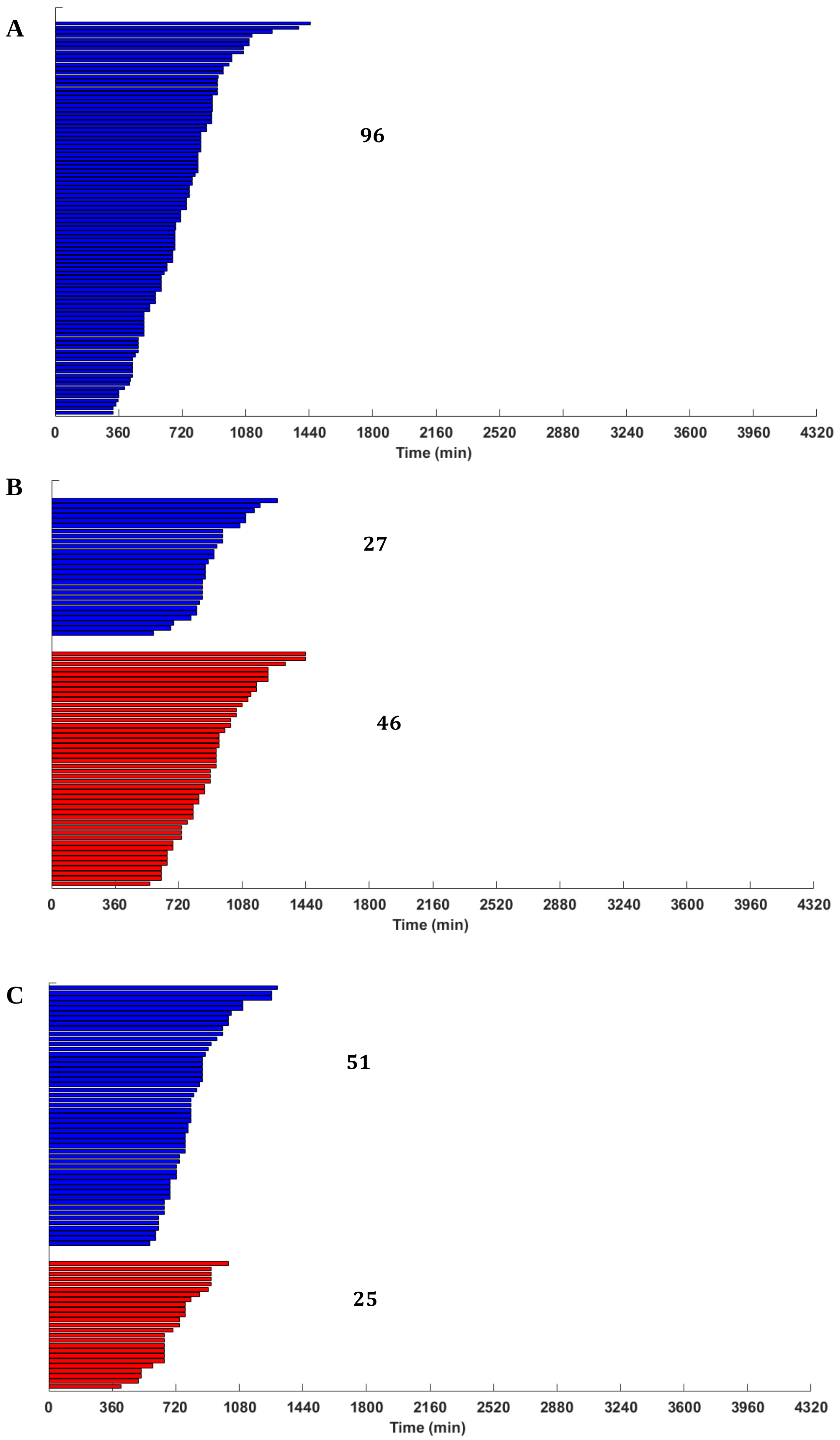

A
96
0
360
720
1080
1440
1800
2160
2520
2880
3240
3600
3960
4320
Time (min)
B
27
46
0
360
720
1080
1440
1800
2160
2520
2880
3240
3600
3960
4320
Time (min)
C
51
25
0
360
720
1080
1440
1800
2160
2520
2880
3240
3600
3960
4320
Time (min)

D

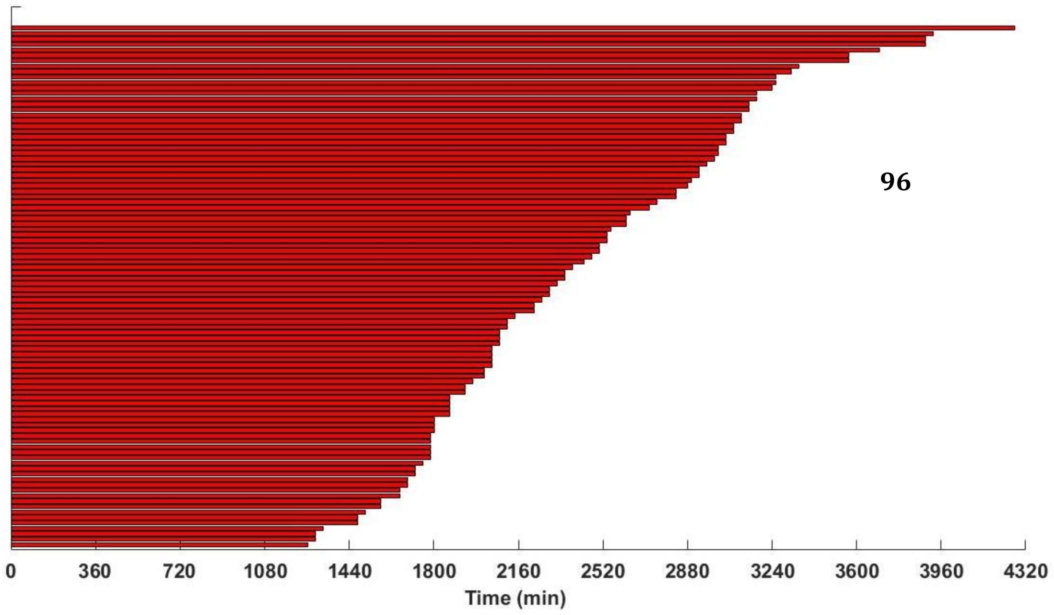
96
0
360
720
1080
1440
1800
2160
2520
2880
3240
3600
3960
4320
Time (min)

E

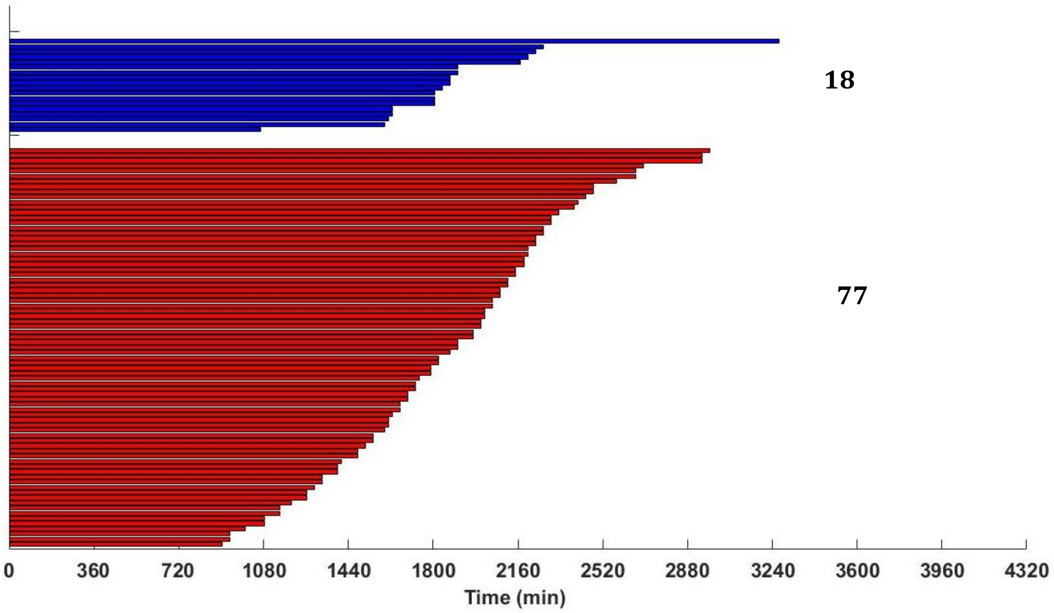
18
77
0
360
720
1080
1440
1800
2160
2520
2880
3240
3600
3960
4320
Time (min)

F

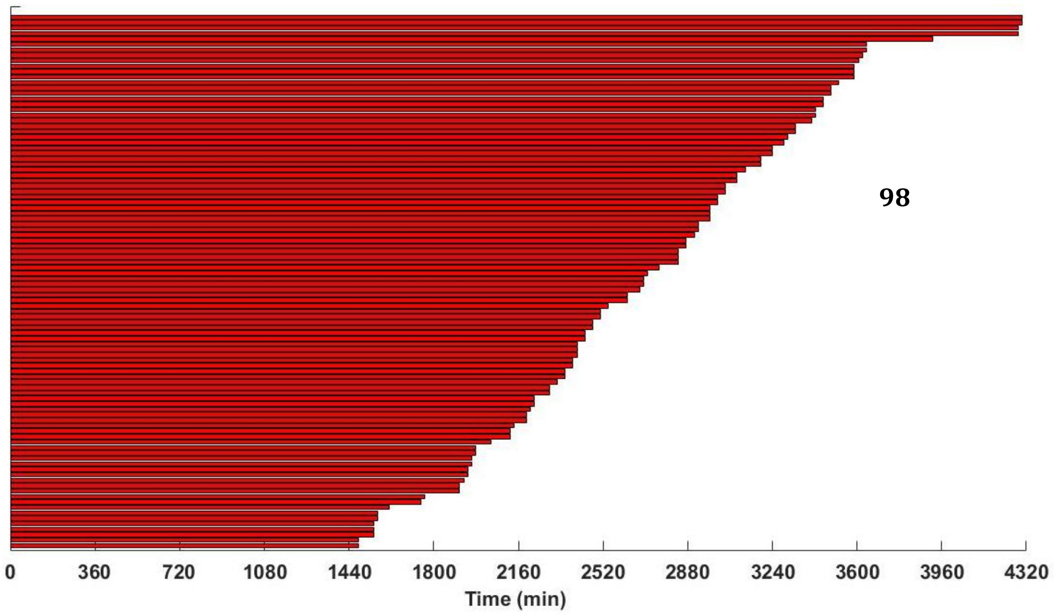
98
0
360
720
1080
1440
1800
2160
2520
2880
3240
3600
3960
4320
Time (min)

G

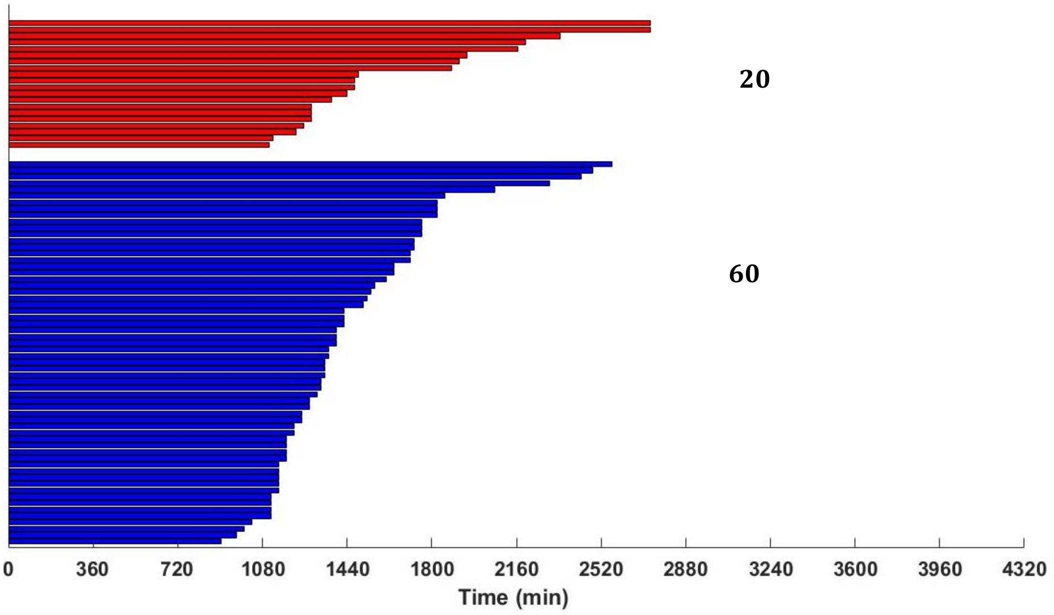
20
60
0
360
720
1080
1440
1800
2160
2520
2880
3240
3600
3960
4320
Time (min)

H

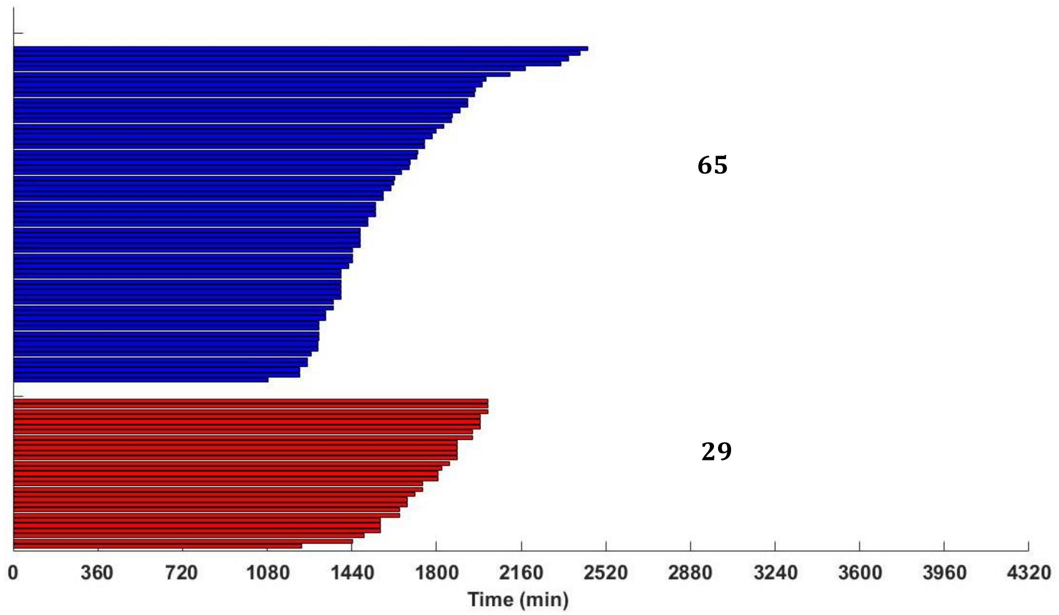
65
29
0
360
720
1080
1440
1800
2160
2520
2880
3240
3600
3960
4320
Time (min)

I

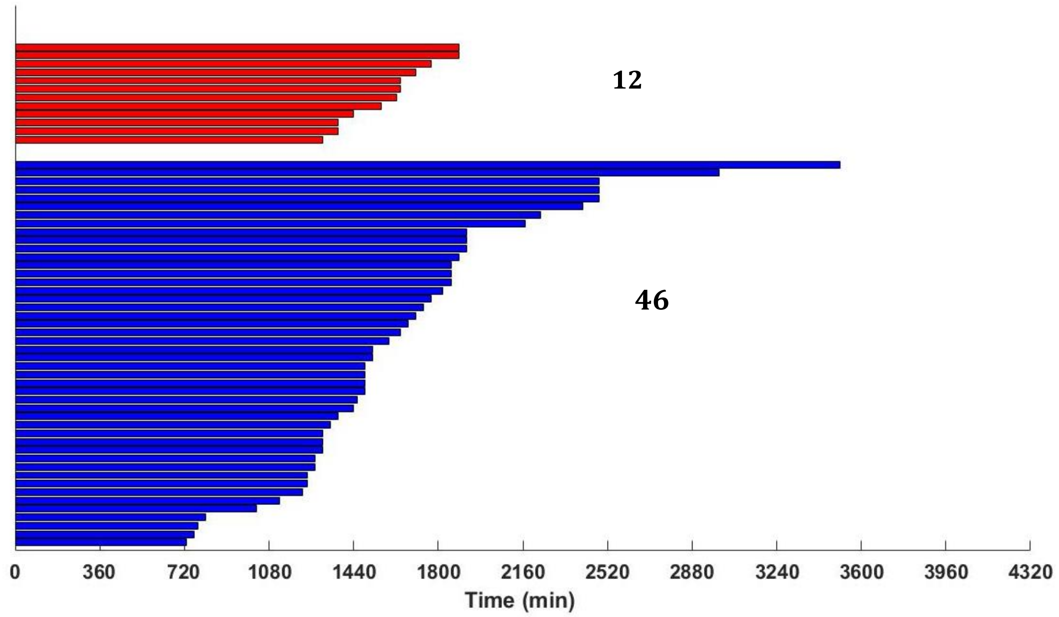
12
46
0
360
720
1080
1440
1800
2160
2520
2880
3240
3600
3960
4320
Time (min)

J

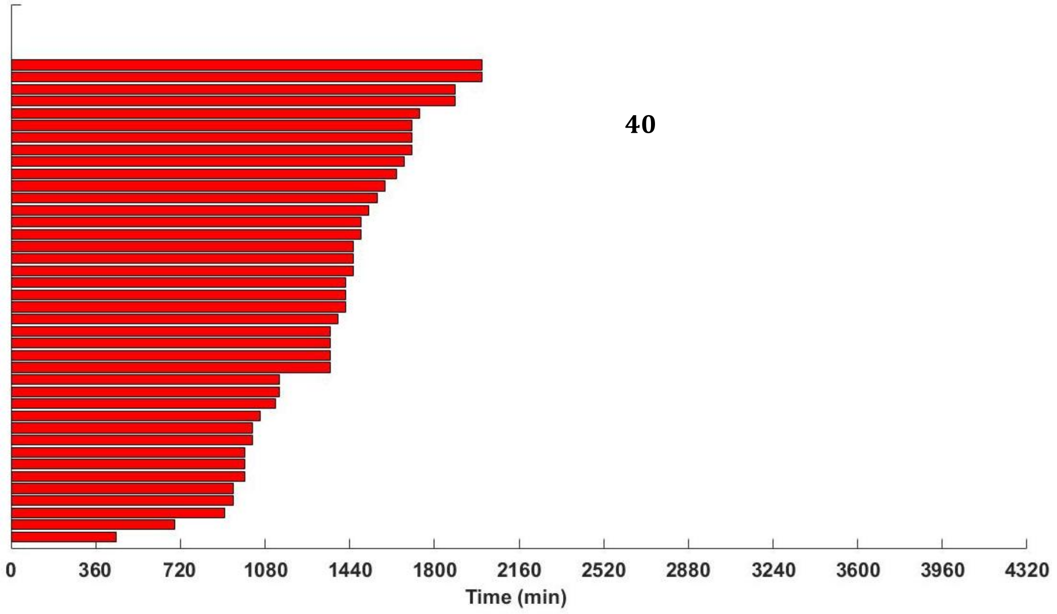
40
0
360
720
1080
1440
1800
2160
2520
2880
3240
3600
3960
4320
Time (min)

K

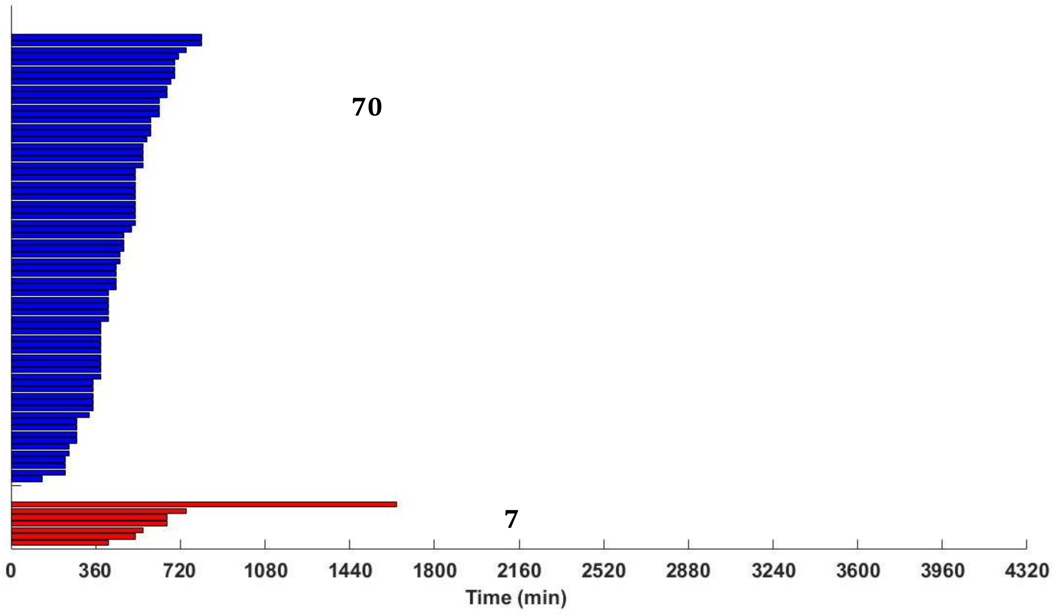
70
7
0
360
720
1080
1440
1800
2160
2520
2880
3240
3600
3960
4320
Time (min)

L

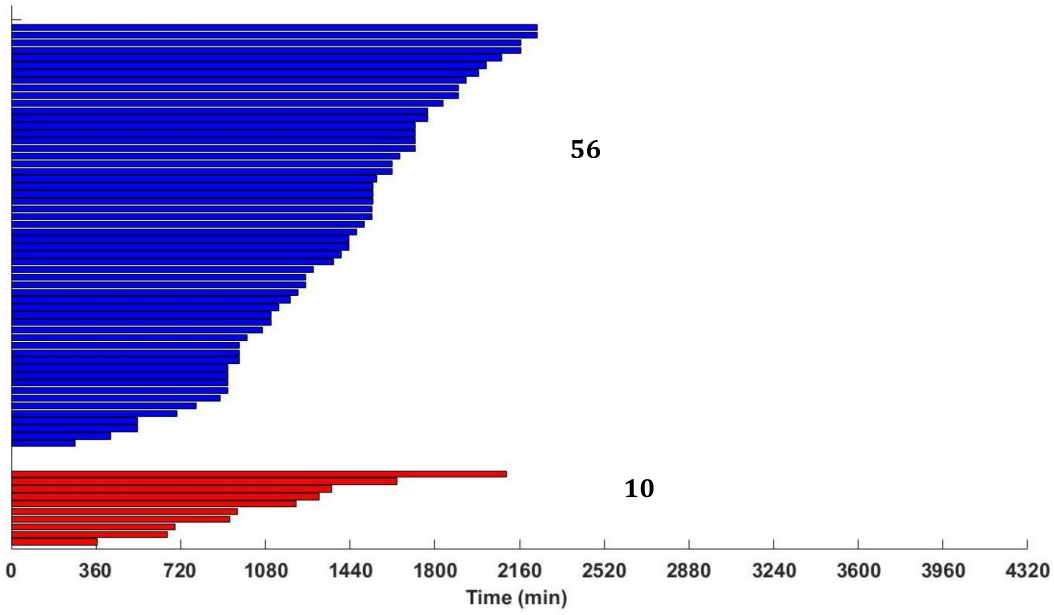

56
10
0
360
720
1080
1440
1800
2160
2520
2880
3240
3600
3960
4320
Time (min)

M

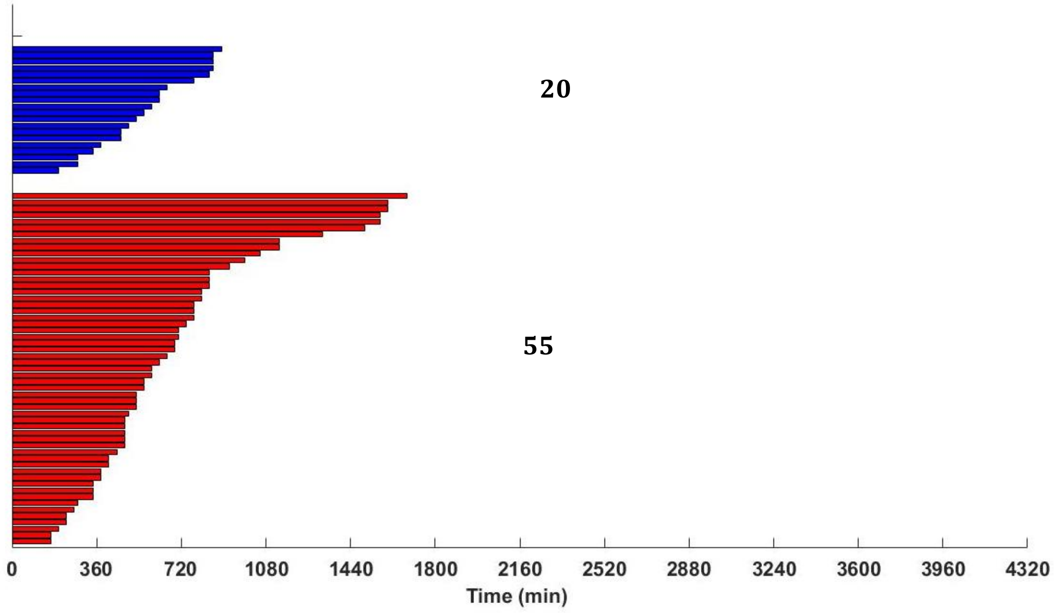

20
55
0
360
720
1080
1440
1800
2160
2520
2880
3240
3600
3960
4320
Time (min)

N

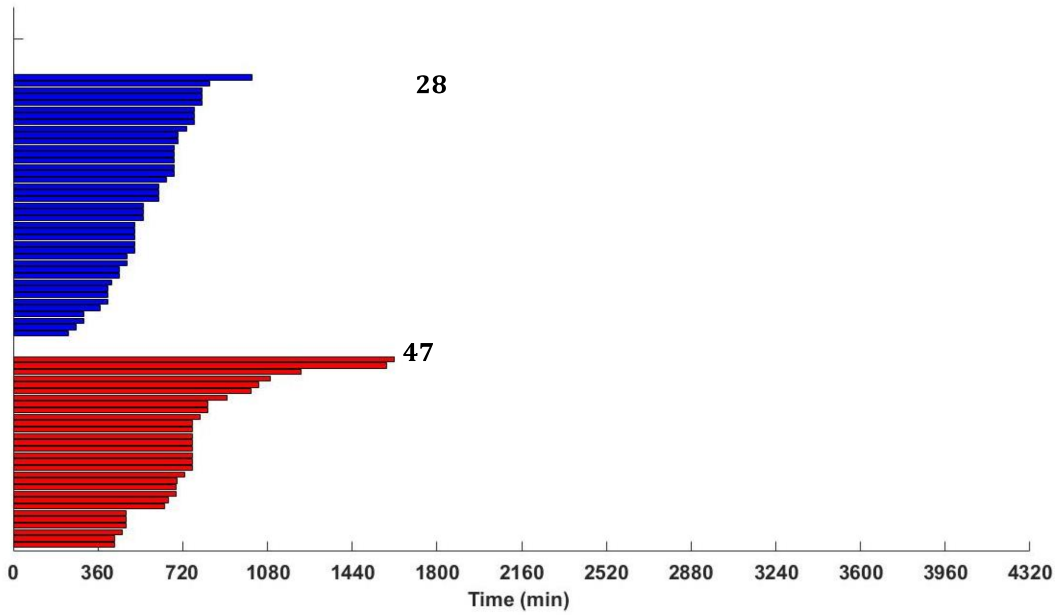
28
47
0
360
720
1080
1440
1800
2160
2520
2880
3240
3600
3960
4320
Time (min)

O

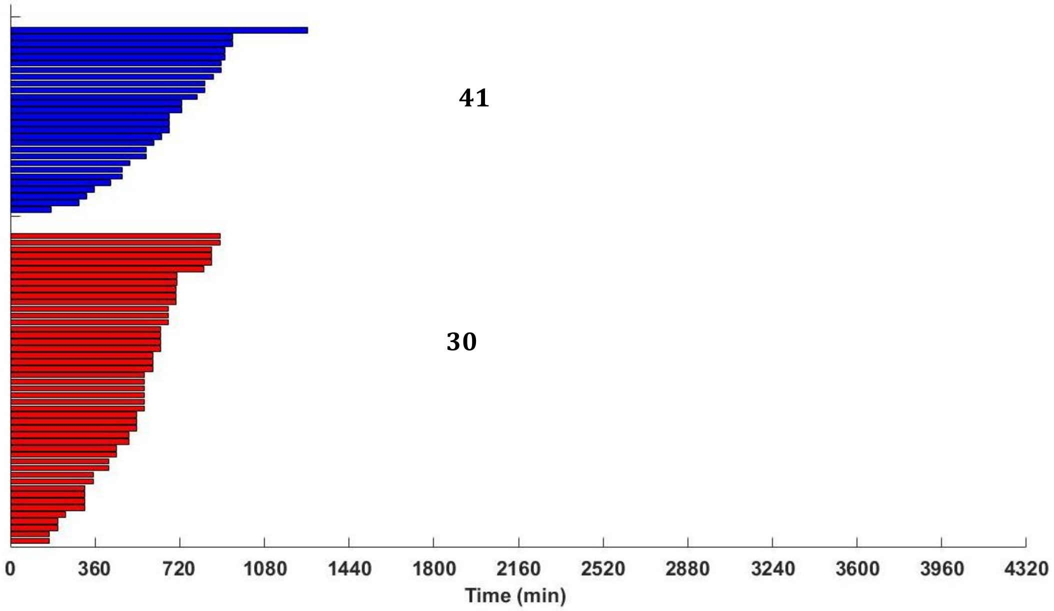
41
30
0
360
720
1080
1440
1800
2160
2520
2880
3240
3600
3960
4320
Time (min)

P

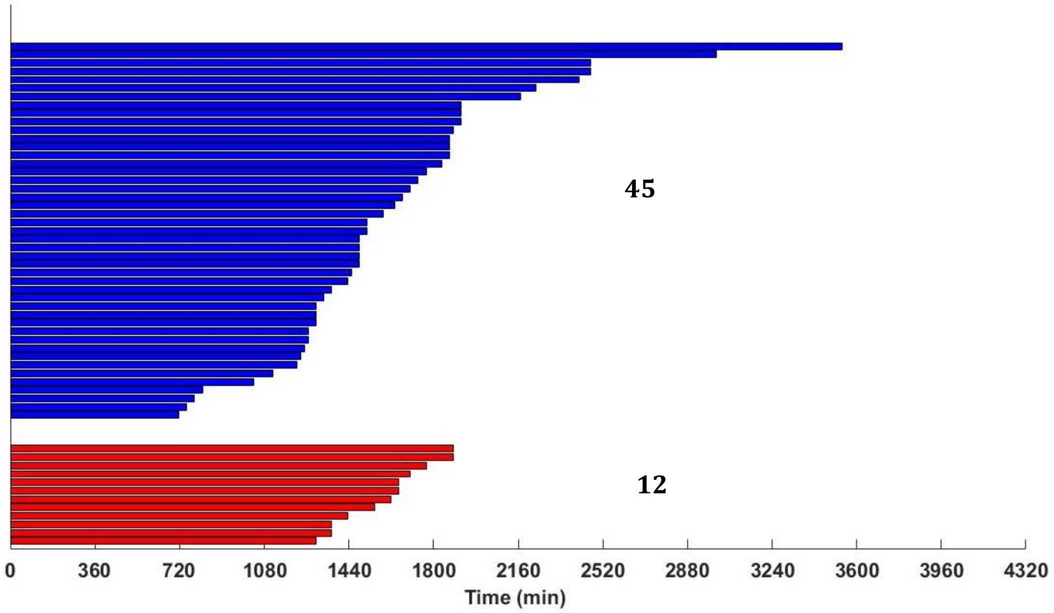

Supplementary Figure 1**. Cell response following prolonged exposure to antimitotic drugs during a 72-hour imaging period.**

RKO cell response to (A) 30 ng/mL nocodazole + 100 μM Boc-D-FMK (a pan-caspase inhibitor), (B) 0.1 μM taxol + 100 μM Boc-D-FMK (a pan-caspase inhibitor), (C) 1 μM AZ138 + 100 μM Boc-D-FMK (a pan-caspase inhibitor), HT29 cell response to (D) 30 ng/mL nocodazole, (E) 0.1 μM taxol, (F) 1 μM AZ138, (G) 30 ng/mL nocodazole + 100 μM Boc-D-FMK (a pan-caspase inhibitor), (H) 0.1 μM taxol + 100 μM Boc-D-FMK (a pan-caspase inhibitor), (I) 1 μM AZ138 + 100 μM Boc-D-FMK (a pan-caspase inhibitor), (J) 0.03 μM AZ138, HCT116 cell response to (K) 30 ng/mL nocodazole, (L) 0.1 μM taxol, (M) 1 μM AZ138, (N) 100 μM monastrol, (O) 1 μM AZ138 + 100 μM Boc-D-FMK (a pan-caspase inhibitor), and (P) 0.03 μM AZ138 during a 72-hour imaging period. Data are adapted from the experimental findings reported in Figure S5A in [1]. Each horizontal bar represents the fate of a single cell. The number of cells corresponding to each category is shown in bold black in each panel.

A

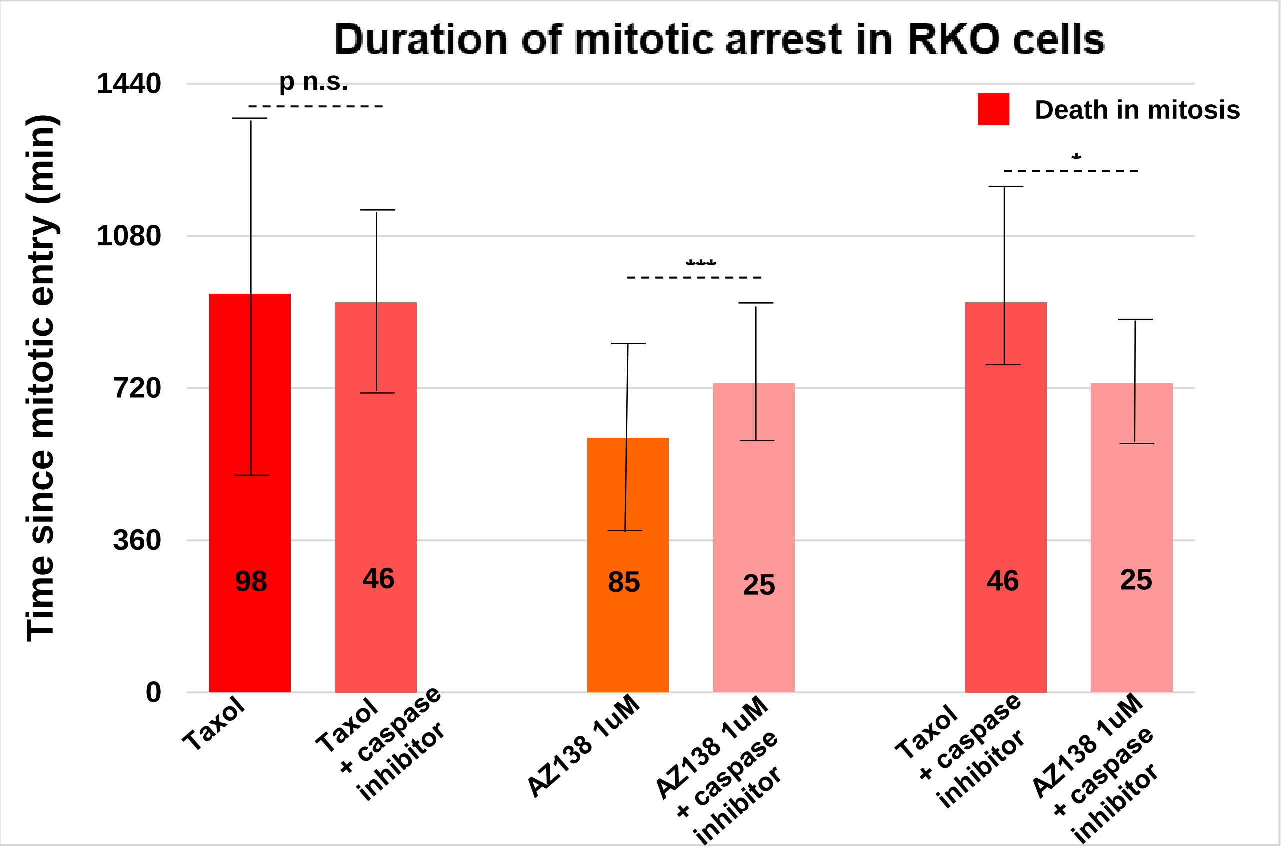
Duration of mitotic arrest in RKO cells
Death in mitosis
Time since mitotic entry (min)
1440
1080
720
360
0
p n.s.
***
*
98
46
85
25
46
25
Taxol
Taxol + caspase inhibitor
AZ138 1uM
AZ138 1uM + caspase inhibitor
Taxol + caspase inhibitor
AZ138 1uM + caspase inhibitor

B

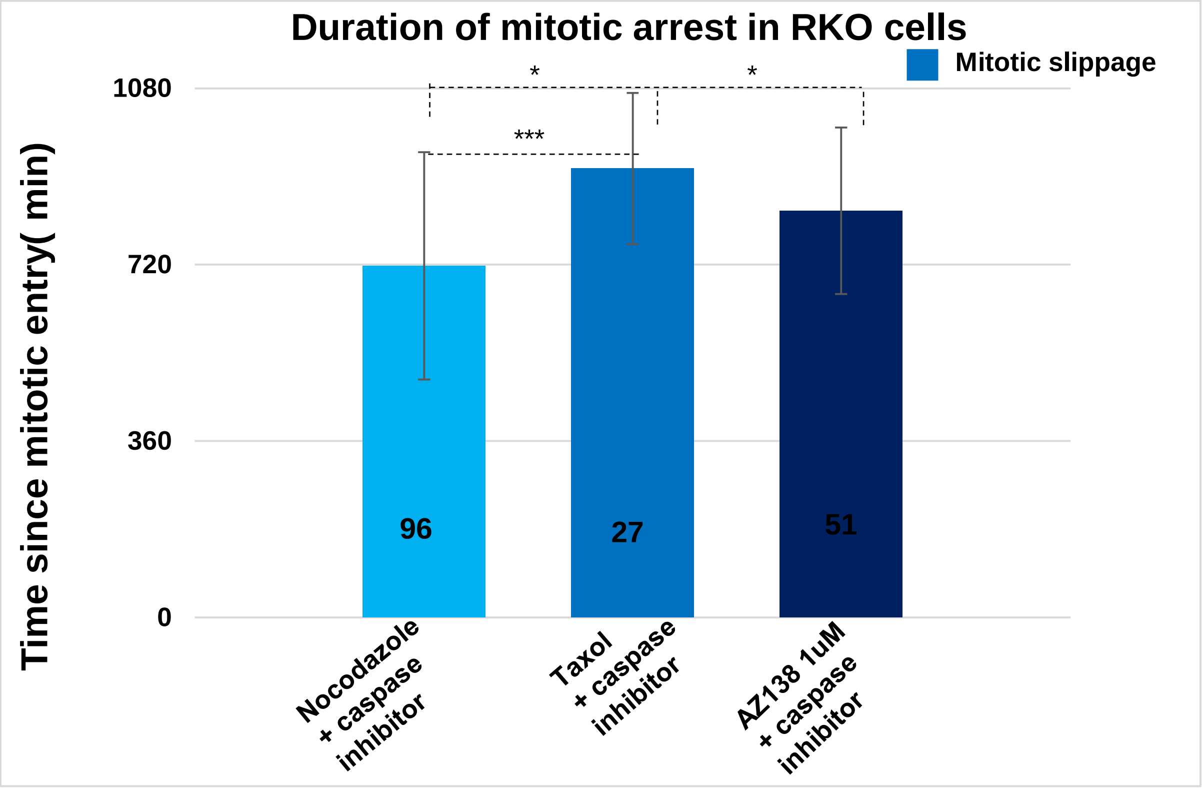
Duration of mitotic arrest in RKO cells
Mitotic slippage
Time since mitotic entry(min)
1080
720
360
0
*
*
***
96
27
51
Nocodazole + caspase inhibitor
Taxol + caspase inhibitor
AZ138 1uM + caspase inhibitor

Supplementary Figure 2. **Duration of mitotic arrest in RKO cells in the presence and absence of the pan-caspase inhibitor Boc-D-FMK.**

Statistical differences between the different RKO cell responses under prolonged exposure to a specific antimitotic drug in the presence and absence of the pan-caspase inhibitor Boc-D-FMK were analyzed using the non-parametric Mann-Whitney test; n.s., non-significant, * $p < 0.01$, ** $p < 0.001$, *** $p < 0.0001$. Pairwise comparisons were performed among all possible combinations between the groups of cells that (A) died in mitosis (red bars) or that (B) underwent mitotic slippage (blue bars). The vertical bar plots represents the mean ± s.d. duration of the drug-induced mitotic arrest in either death in mitosis (red bars) or mitotic slippage (blue bars). The reported values are in minutes. The number of cells corresponding to each category is shown in bold black inside each vertical bar plot.

A

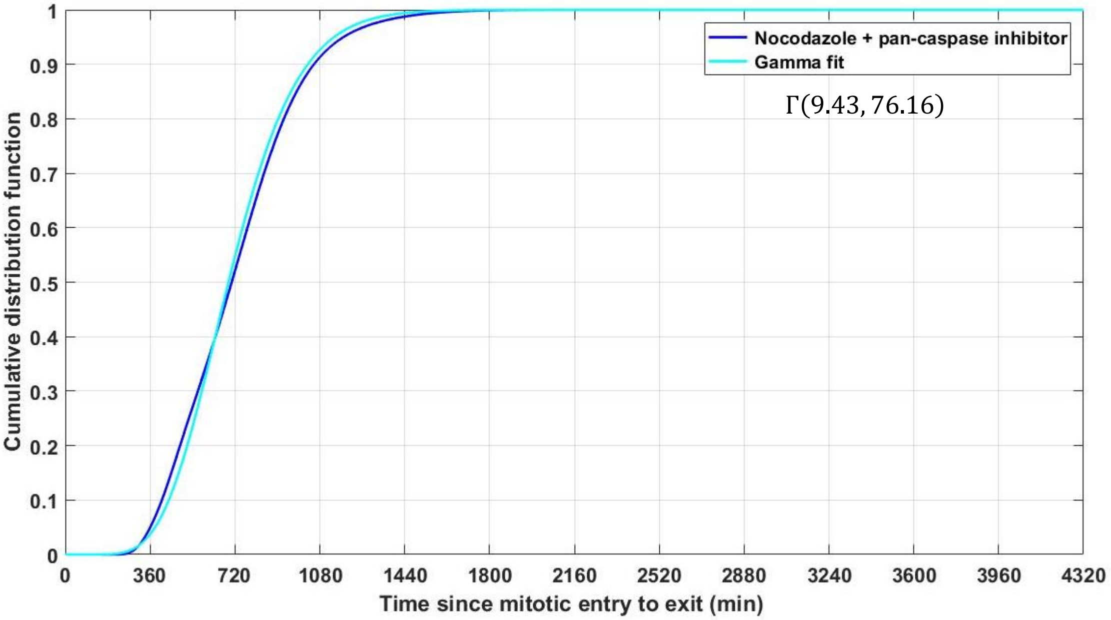

Nocodazole + pan-caspase inhibitor
Gamma fit
Γ(9.43, 76.16)
Cumulative distribution function
Time since mitotic entry to exit (min)

B

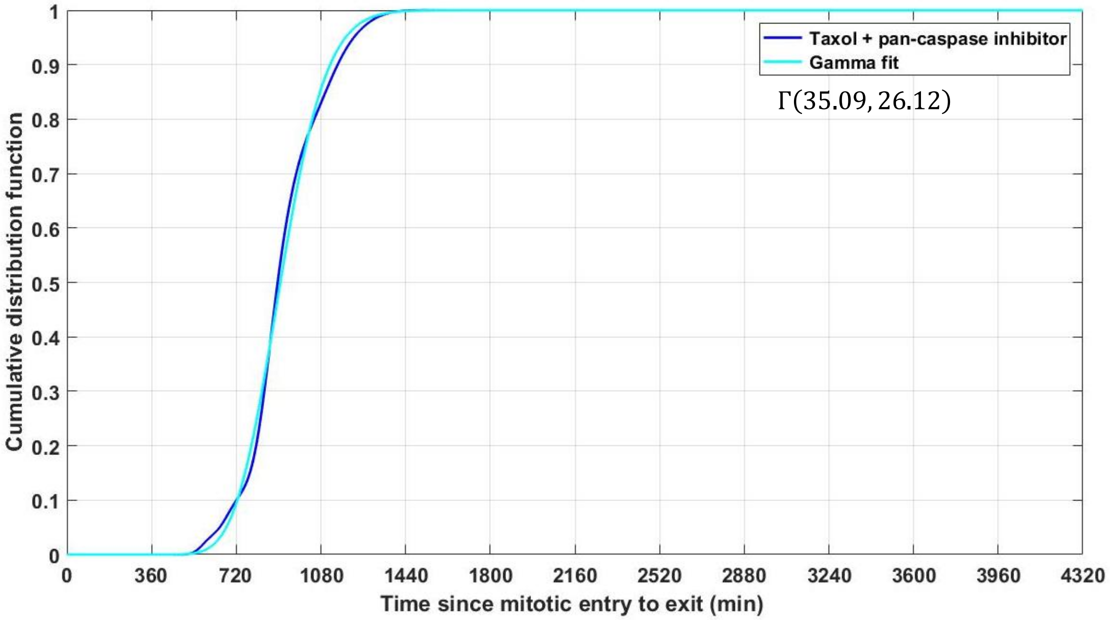

Taxol + pan-caspase inhibitor
Gamma fit
Γ(35.09, 26.12)
Cumulative distribution function
Time since mitotic entry to exit (min)

C

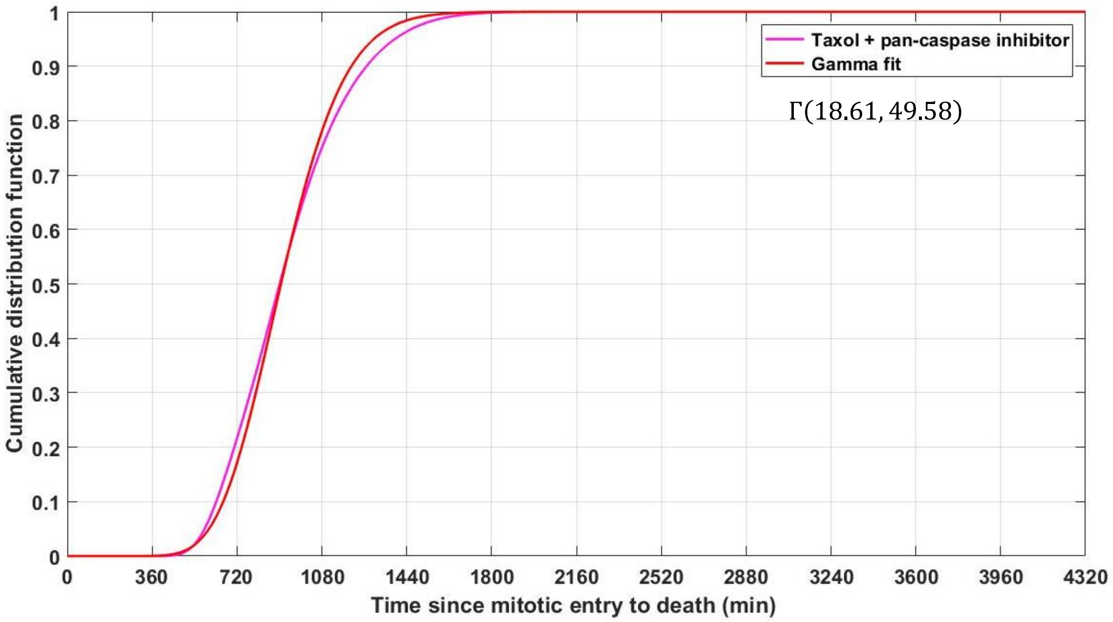

Taxol + pan-caspase inhibitor
Gamma fit
Γ(18.61, 49.58)
Cumulative distribution function
Time since mitotic entry to death (min)
0
360
720
1080
1440
1800
2160
2520
2880
3240
3600
3960
4320

D

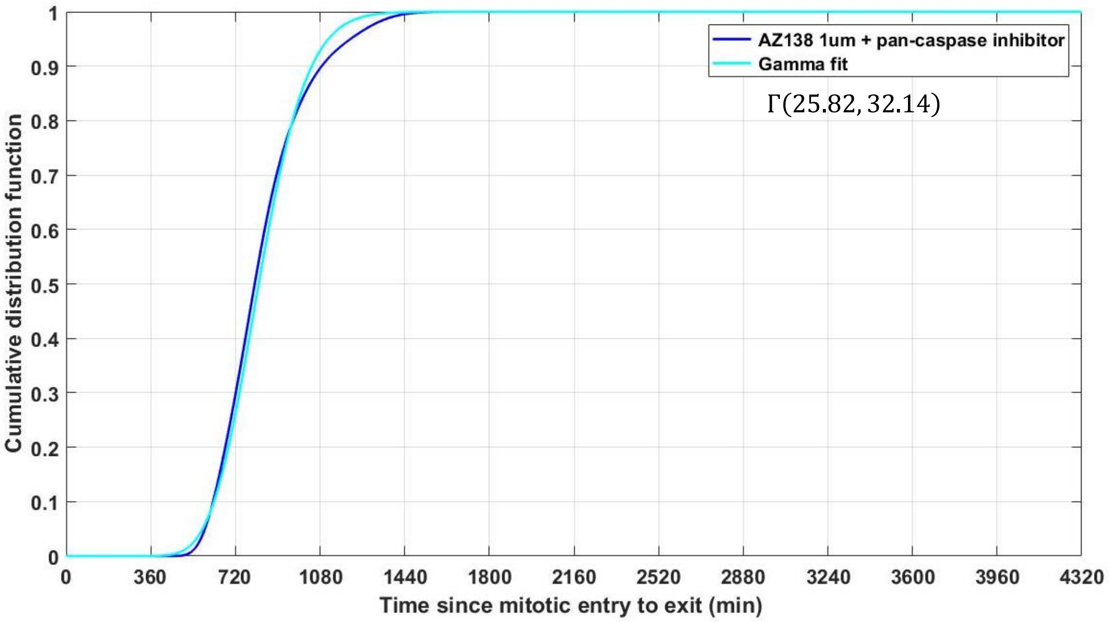

AZ138 1um + pan-caspase inhibitor
Gamma fit
Γ(25.82, 32.14)
Cumulative distribution function
Time since mitotic entry to exit (min)
0
360
720
1080
1440
1800
2160
2520
2880
3240
3600
3960
4320

E

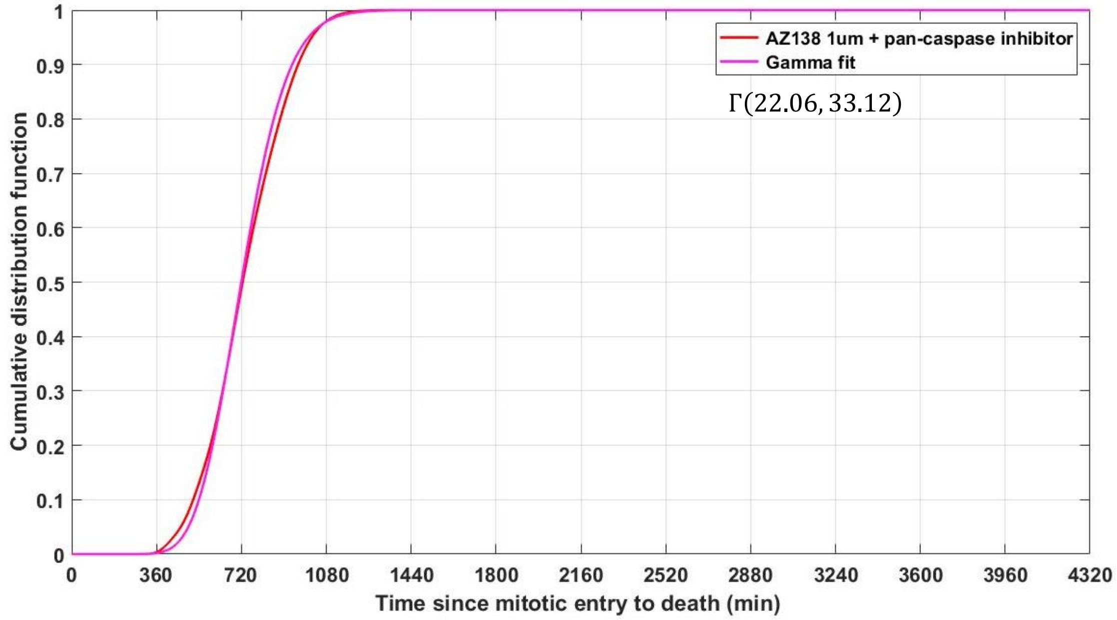

AZ138 1um + pan-caspase inhibitor
Gamma fit
Γ(22.06, 33.12)
Cumulative distribution function
Time since mitotic entry to death (min)

F

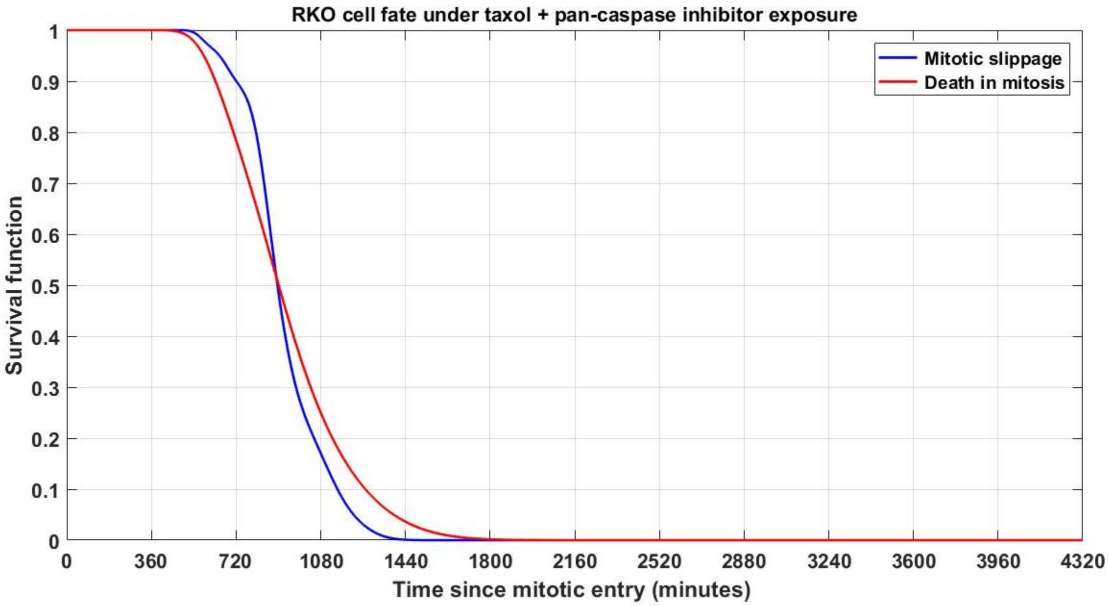

RKO cell fate under taxol + pan-caspase inhibitor exposure
Mitotic slippage
Death in mitosis
Survival function
Time since mitotic entry (minutes)

G

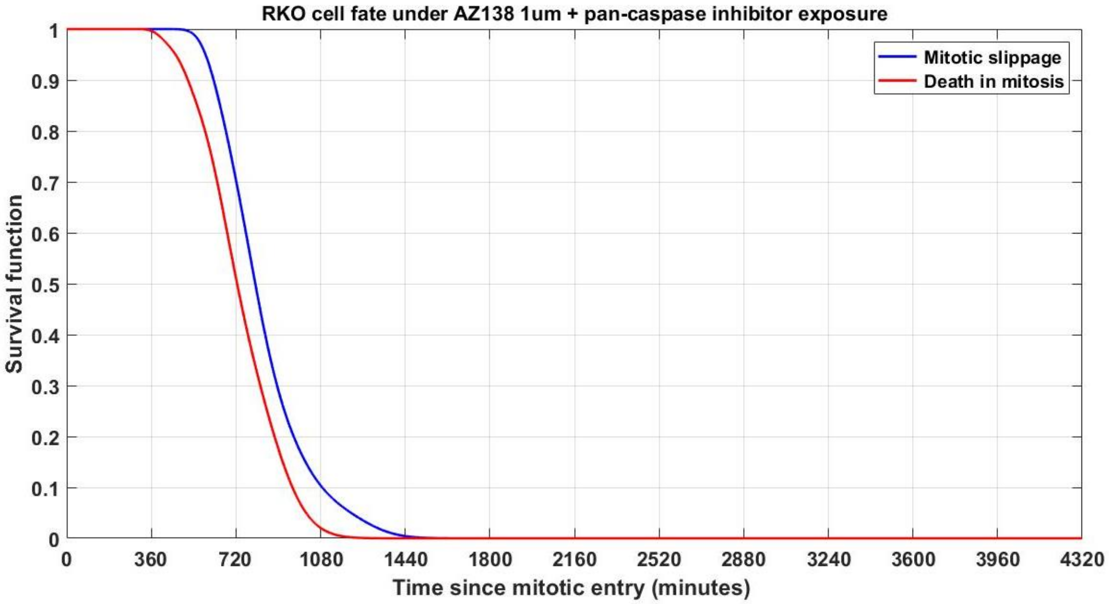

Supplementary Figure 3. **RKO cell death and slippage responses across drugs can be characterized by Gamma distributions even in the presence of the pan-caspase inhibitor Boc-D-FMK.**

The cumulative distribution functions (CDF) for the death in mitosis and mitotic slippage show the fraction of RKO cells that either died or slipped after entering mitosis as a function of time. Data are adapted from the experimental findings reported in Figure S5A in [1] in the presence of the pan-caspase inhibitor Boc-D-FMK. Therein, the RKO cellular fate following prolonged exposure to four different drugs (monastrol, nocodazole, taxol and AZ138) was measured, based on the duration of drug-induced mitotic arrest. Cell death or slippage responses across drugs in panels (A) – (E)can be characterized by the cell-cycle age "a"-dependent Gamma distribution $\Gamma(a; k, \theta)$, with shape parameter k and scale parameter θ. For each panel, we report the corresponding Gamma CDF parameters in the inset adjacent to the figure legend. We additionally report the RKO cell fate in the death in mitosis and mitotic slippage pathways as CDFs under (F) 30ng/ml nocodazole and (G) 0.1 µM taxol prolonged exposure in the presence of the pan-caspase inhibitor Boc-D-FMK.

A

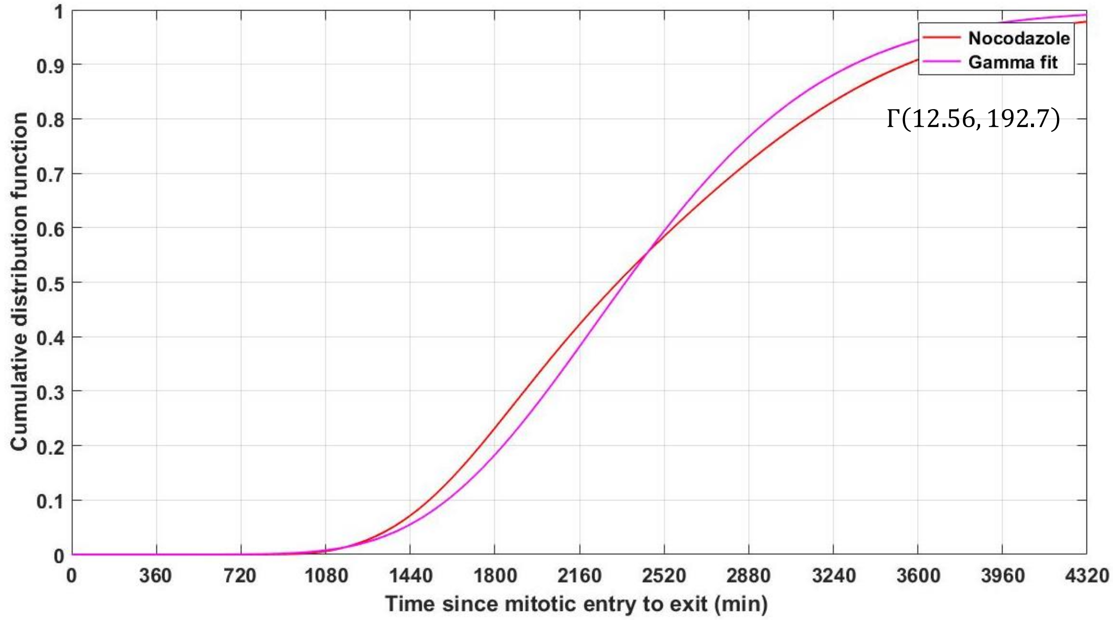
Nocodazole
Gamma fit
Γ(12.56, 192.7)
Cumulative distribution function
Time since mitotic entry to exit (min)
0
360
720
1080
1440
1800
2160
2520
2880
3240
3600
3960
4320
0.1
0.2
0.3
0.4
0.5
0.6
0.7
0.8
0.9
1

B

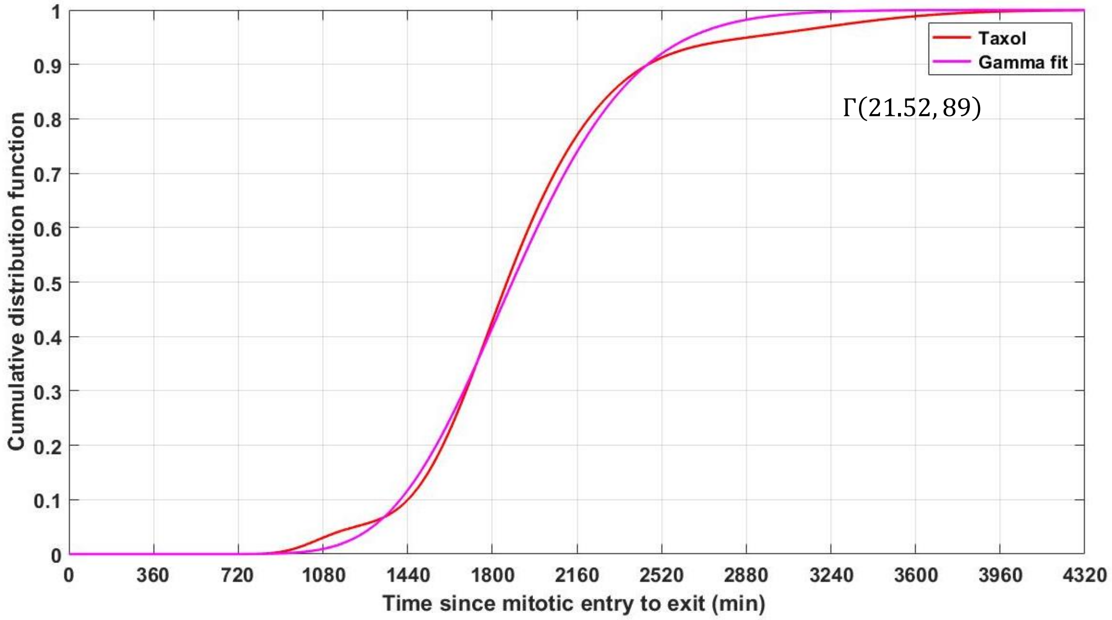
Taxol
Gamma fit
Γ(21.52, 89)
Cumulative distribution function
Time since mitotic entry to exit (min)
0
360
720
1080
1440
1800
2160
2520
2880
3240
3600
3960
4320
0.1
0.2
0.3
0.4
0.5
0.6
0.7
0.8
0.9
1

C

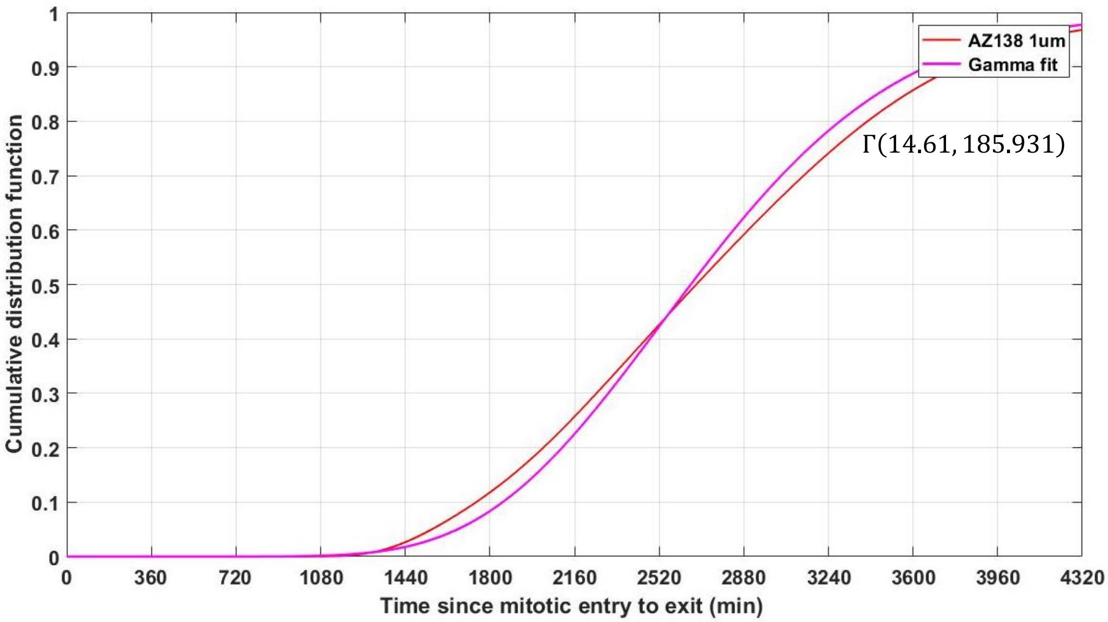
AZ138 1um
Gamma fit
Γ(14.61, 185.931)
Cumulative distribution function
Time since mitotic entry to exit (min)
0
0.1
0.2
0.3
0.4
0.5
0.6
0.7
0.8
0.9
1
0
360
720
1080
1440
1800
2160
2520
2880
3240
3600
3960
4320

D

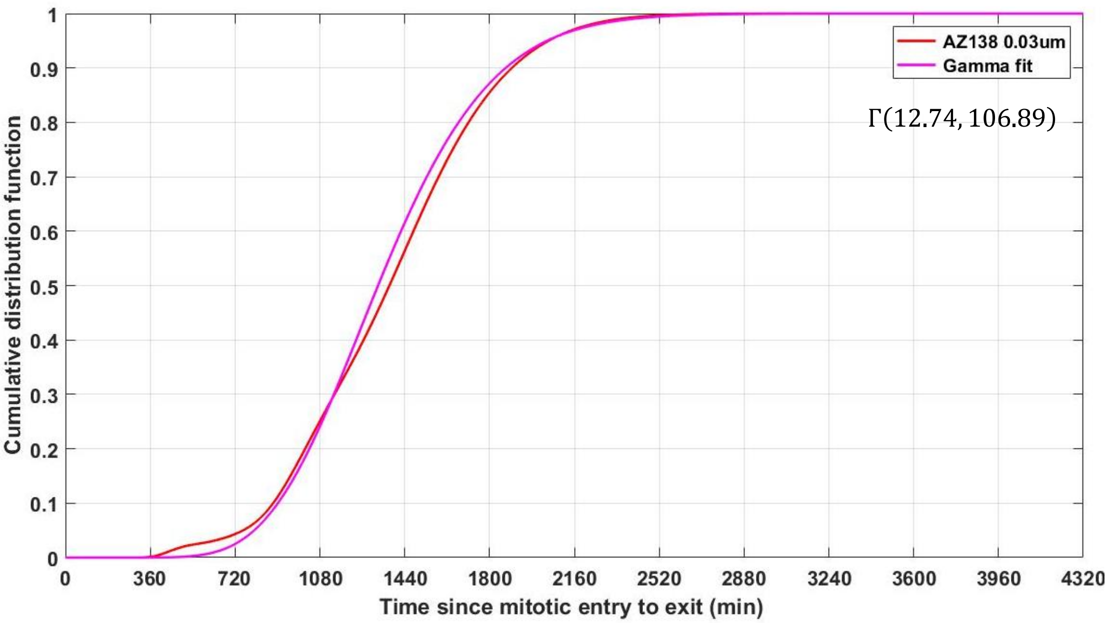
AZ138 0.03um
Gamma fit
Γ(12.74, 106.89)
Cumulative distribution function
Time since mitotic entry to exit (min)
0
0.1
0.2
0.3
0.4
0.5
0.6
0.7
0.8
0.9
1
0
360
720
1080
1440
1800
2160
2520
2880
3240
3600
3960
4320

E

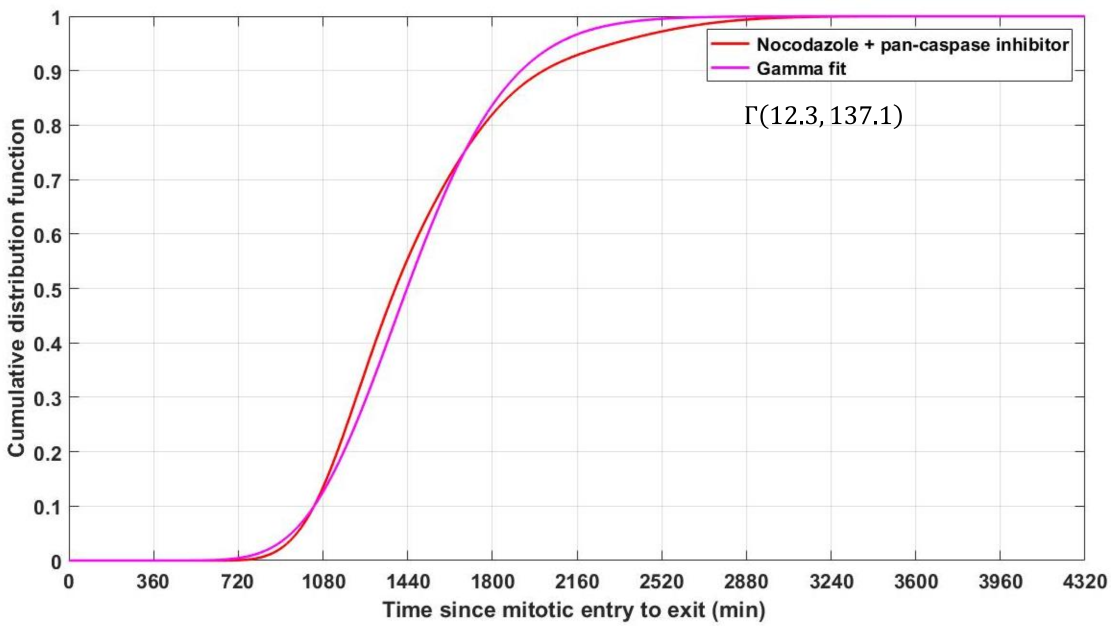

Nocodazole + pan-caspase inhibitor
Gamma fit
$\Gamma(12.3, 137.1)$
Cumulative distribution function
Time since mitotic entry to exit (min)
0
0.1
0.2
0.3
0.4
0.5
0.6
0.7
0.8
0.9
1
0
360
720
1080
1440
1800
2160
2520
2880
3240
3600
3960
4320

F

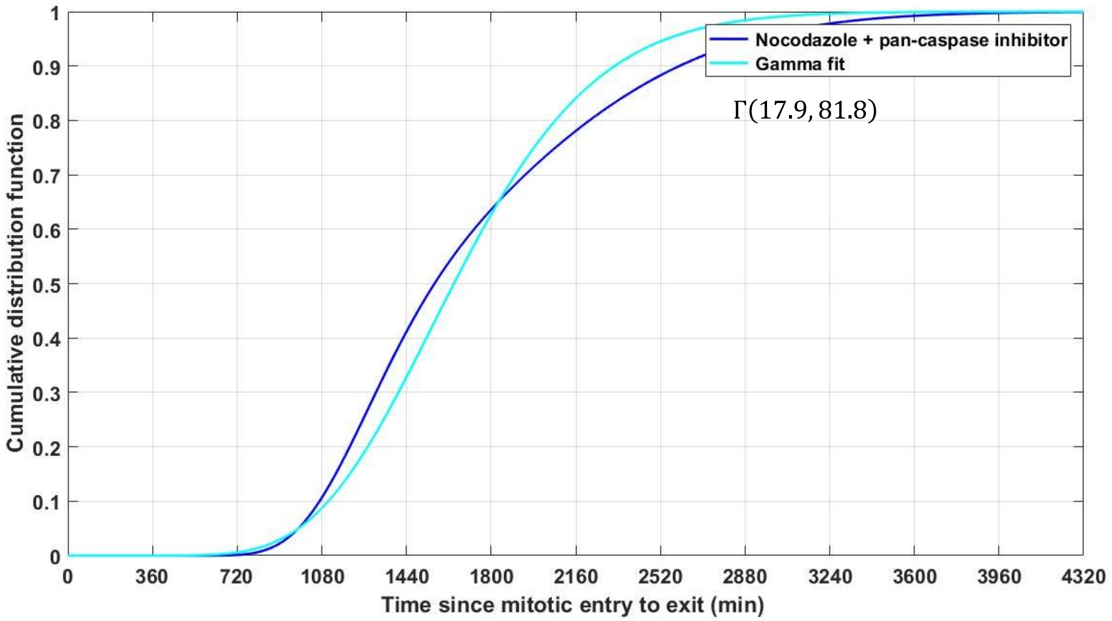

Nocodazole + pan-caspase inhibitor
Gamma fit
$\Gamma(17.9, 81.8)$
Cumulative distribution function
Time since mitotic entry to exit (min)
0
0.1
0.2
0.3
0.4
0.5
0.6
0.7
0.8
0.9
1
0
360
720
1080
1440
1800
2160
2520
2880
3240
3600
3960
4320

G

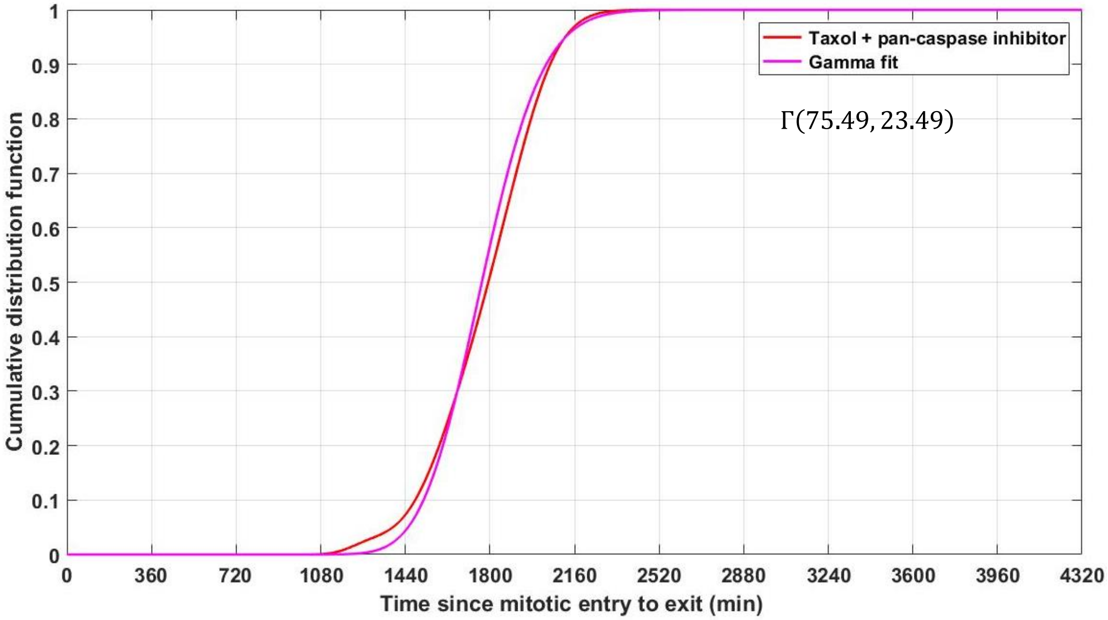
Taxol + pan-caspase inhibitor
Gamma fit
Γ(75.49, 23.49)
Cumulative distribution function
Time since mitotic entry to exit (min)
0
0.1
0.2
0.3
0.4
0.5
0.6
0.7
0.8
0.9
1
0
360
720
1080
1440
1800
2160
2520
2880
3240
3600
3960
4320

H

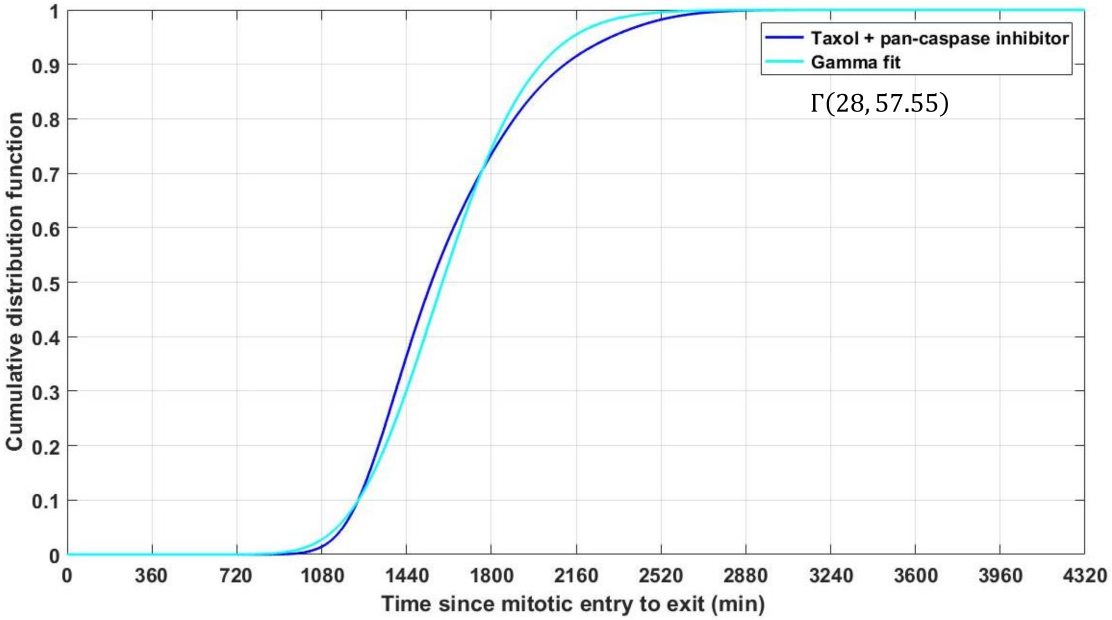
Taxol + pan-caspase inhibitor
Gamma fit
Γ(28, 57.55)
Cumulative distribution function
Time since mitotic entry to exit (min)
0
0.1
0.2
0.3
0.4
0.5
0.6
0.7
0.8
0.9
1
0
360
720
1080
1440
1800
2160
2520
2880
3240
3600
3960
4320

I

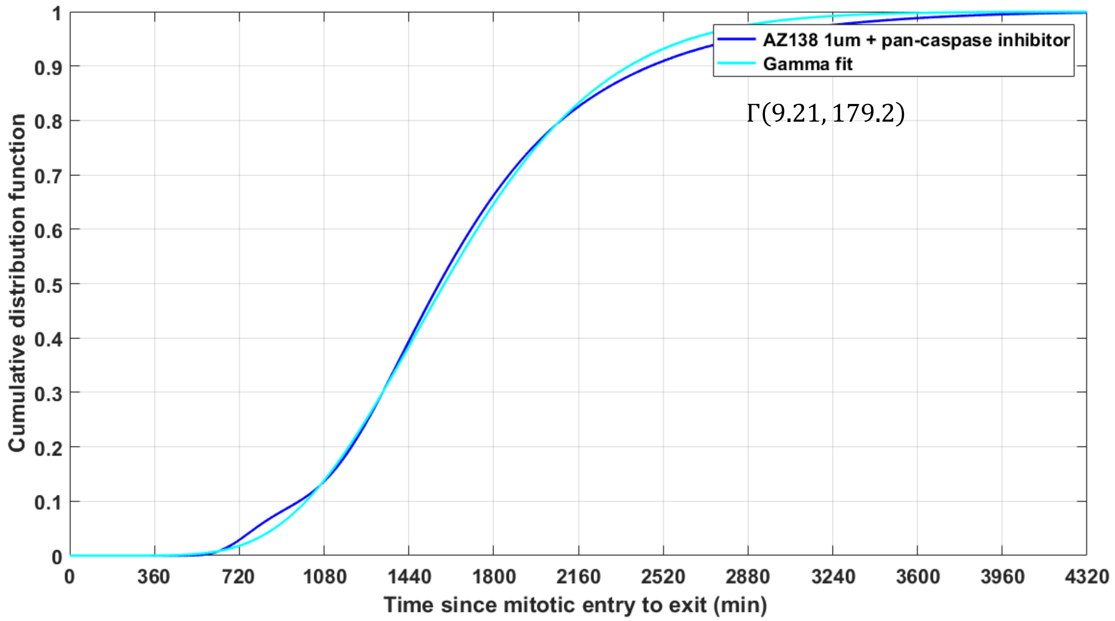

Supplementary Figure 4. **HT29 cell death and slippage responses across drugs can be characterized by Gamma distributions even in the presence of the pan-caspase inhibitor Boc-D-FMK.**

The cumulative distribution functions (CDF) for the death in mitosis and mitotic slippage show the fraction of RKO cells that either died or slipped after entering mitosis as a function of time. Data are adapted from the experimental findings reported in Figure S5A in [1] in the presence of the pan-caspase inhibitor Boc-D-FMK. Therein, the HT29 cellular fate following prolonged exposure to four different drugs (monastrol, nocodazole, taxol and AZ138) was measured, based on the duration of drug-induced mitotic arrest. Cell death or slippage responses across drugs in panels (A) – (I) can be characterized by the cell-cycle age "a"-dependent Gamma distribution $\Gamma(a; k, \theta)$, with shape parameter k and scale parameter θ. For each panel, we report the corresponding Gamma CDF parameters in the inset adjacent to the figure legend.

A

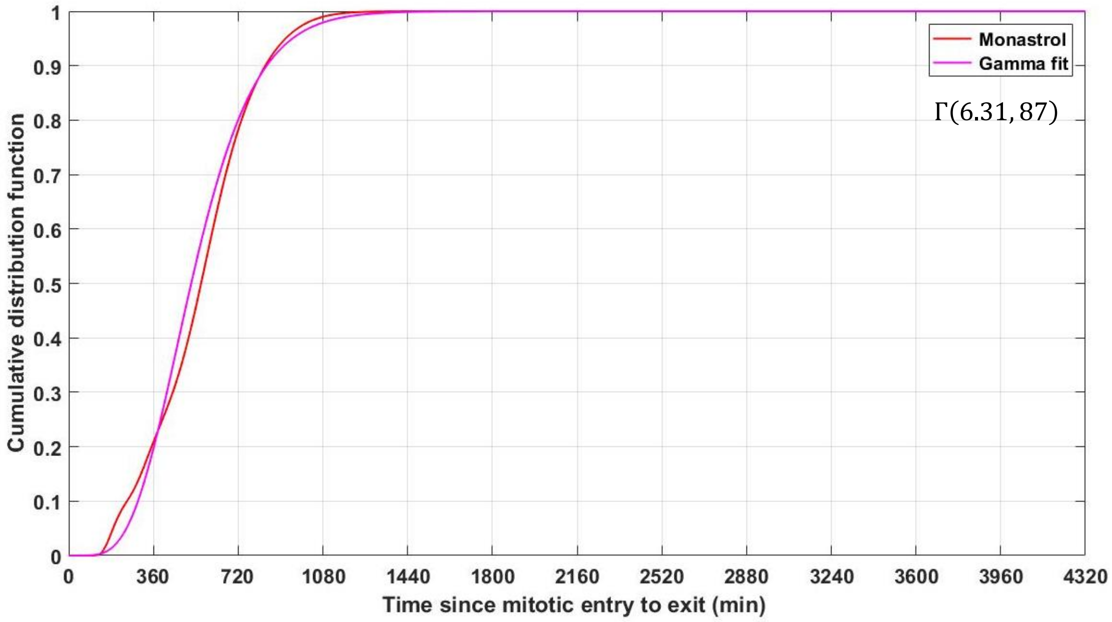
Monastrol
Gamma fit
Γ(6.31, 87)
Cumulative distribution function
Time since mitotic entry to exit (min)
0
0.1
0.2
0.3
0.4
0.5
0.6
0.7
0.8
0.9
1
0
360
720
1080
1440
1800
2160
2520
2880
3240
3600
3960
4320

B

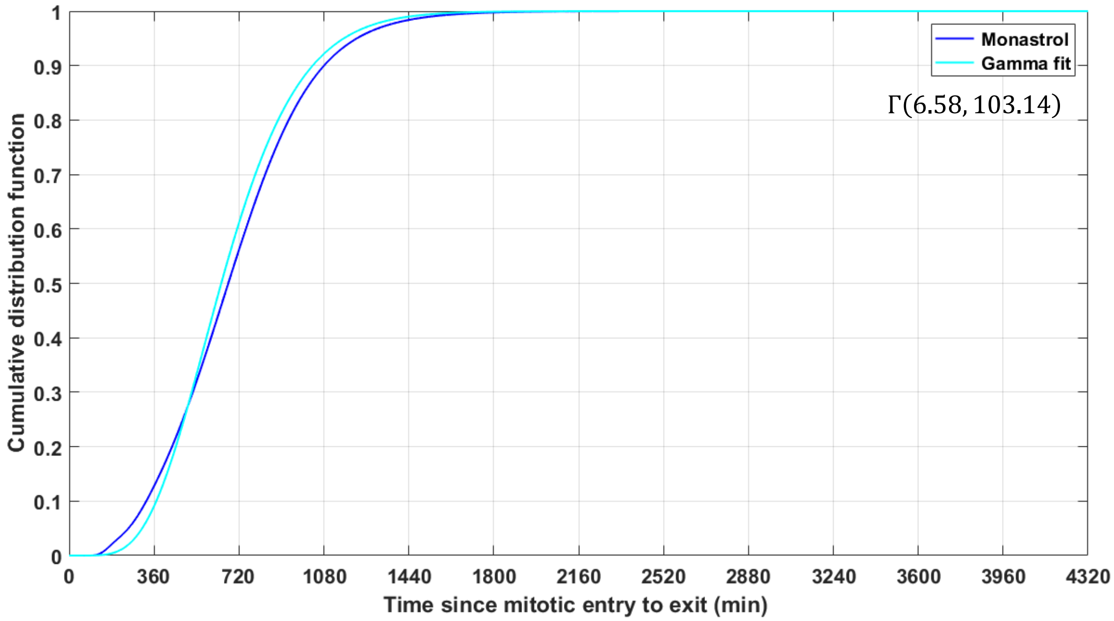
Monastrol
Gamma fit
Γ(6.58, 103.14)
Cumulative distribution function
Time since mitotic entry to exit (min)
0
0.1
0.2
0.3
0.4
0.5
0.6
0.7
0.8
0.9
1
0
360
720
1080
1440
1800
2160
2520
2880
3240
3600
3960
4320

C

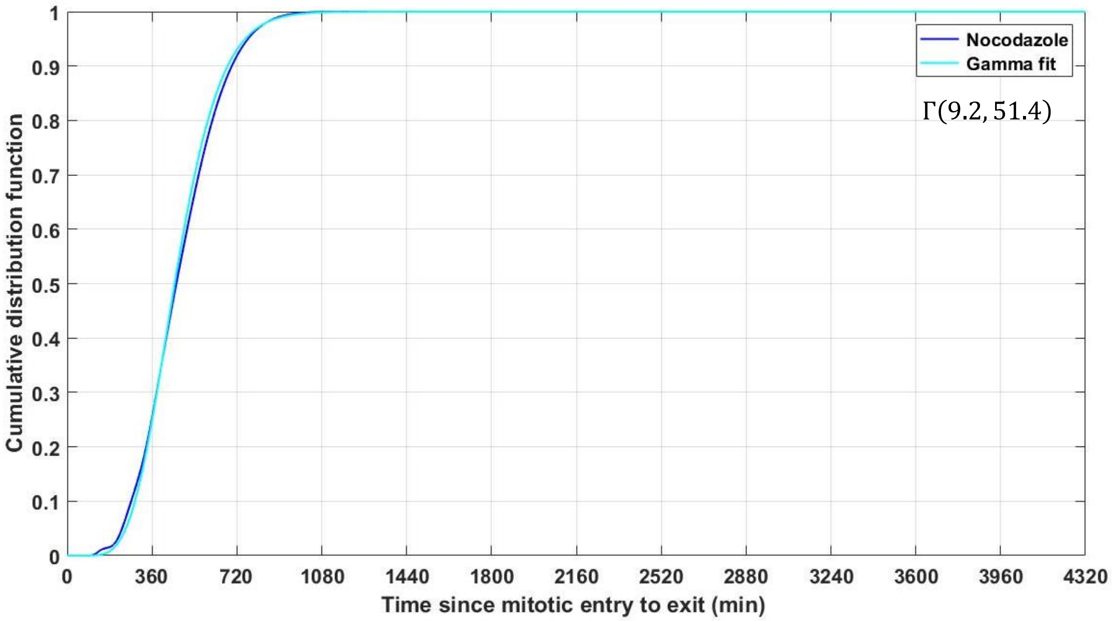

Nocodazole
Gamma fit
Γ(9.2, 51.4)
Cumulative distribution function
Time since mitotic entry to exit (min)

D

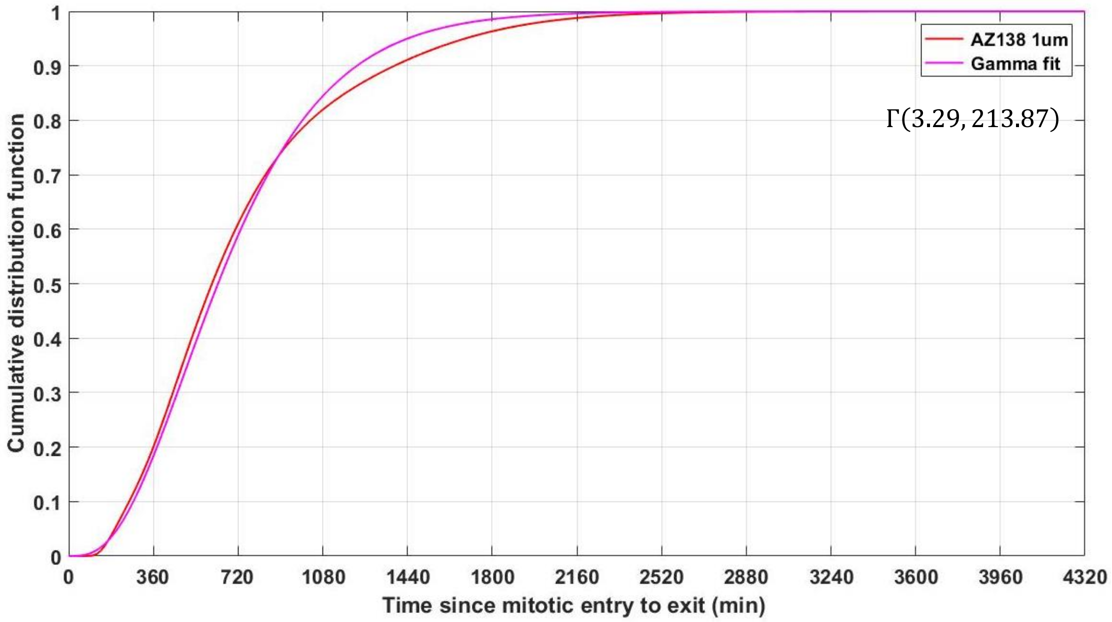

AZ138 1um
Gamma fit
Γ(3.29, 213.87)
Cumulative distribution function
Time since mitotic entry to exit (min)

E

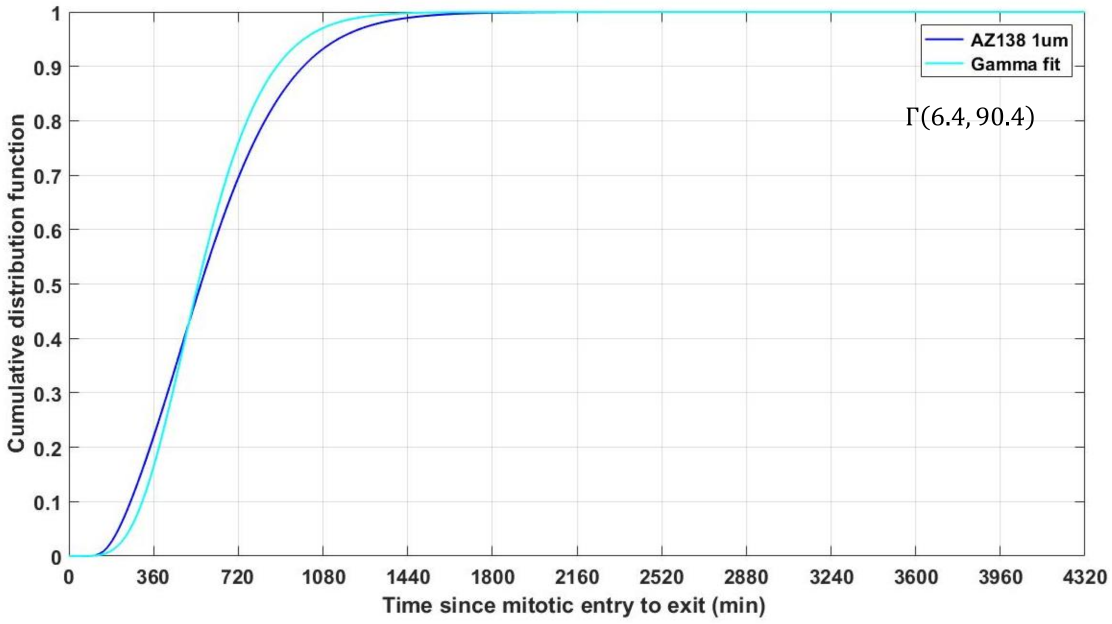
AZ138 1um
Gamma fit
Γ(6.4, 90.4)
Cumulative distribution function
1
0.9
0.8
0.7
0.6
0.5
0.4
0.3
0.2
0.1
0
0 360 720 1080 1440 1800 2160 2520 2880 3240 3600 3960 4320
Time since mitotic entry to exit (min)

F

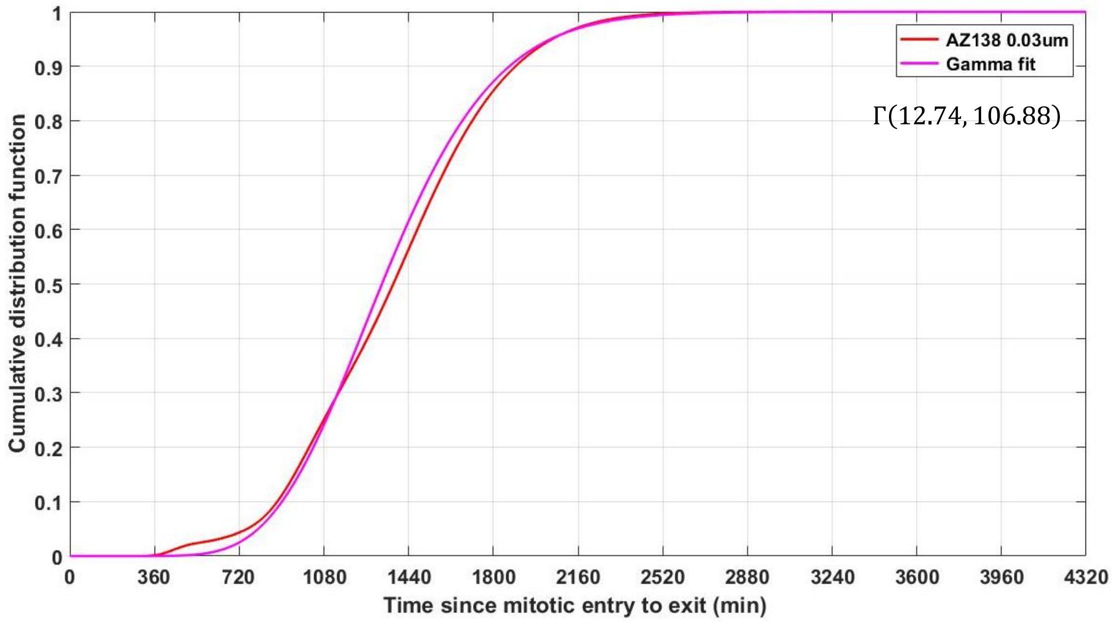
AZ138 0.03um
Gamma fit
Γ(12.74, 106.88)
Cumulative distribution function
1
0.9
0.8
0.7
0.6
0.5
0.4
0.3
0.2
0.1
0
0 360 720 1080 1440 1800 2160 2520 2880 3240 3600 3960 4320
Time since mitotic entry to exit (min)

G

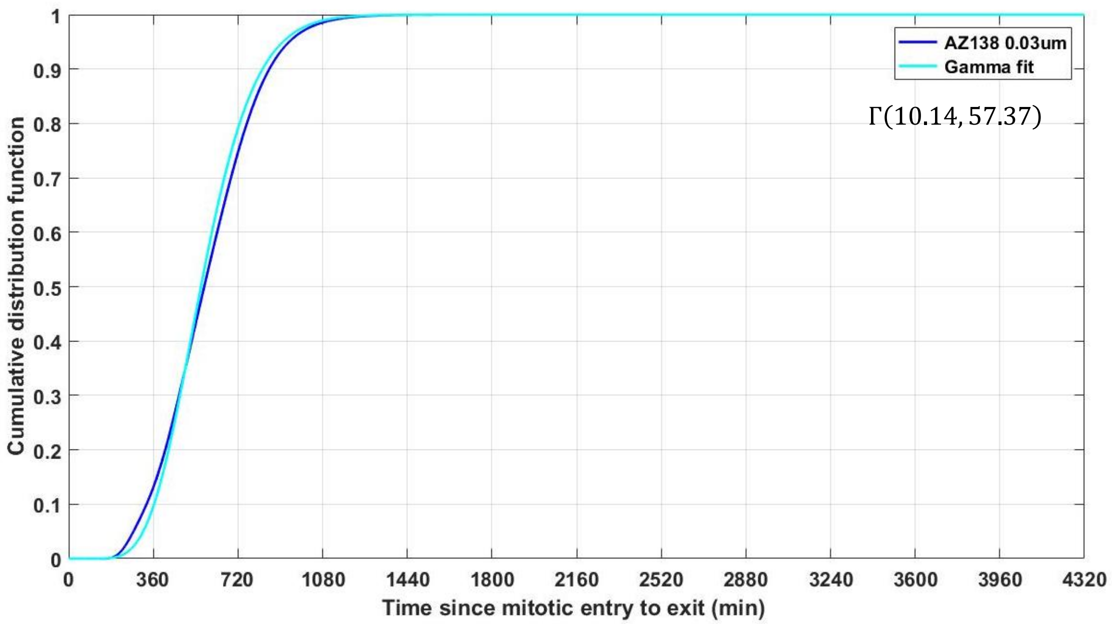
AZ138 0.03um
Gamma fit
Γ(10.14, 57.37)
Cumulative distribution function
Time since mitotic entry to exit (min)
0
0.1
0.2
0.3
0.4
0.5
0.6
0.7
0.8
0.9
1
0
360
720
1080
1440
1800
2160
2520
2880
3240
3600
3960
4320

H

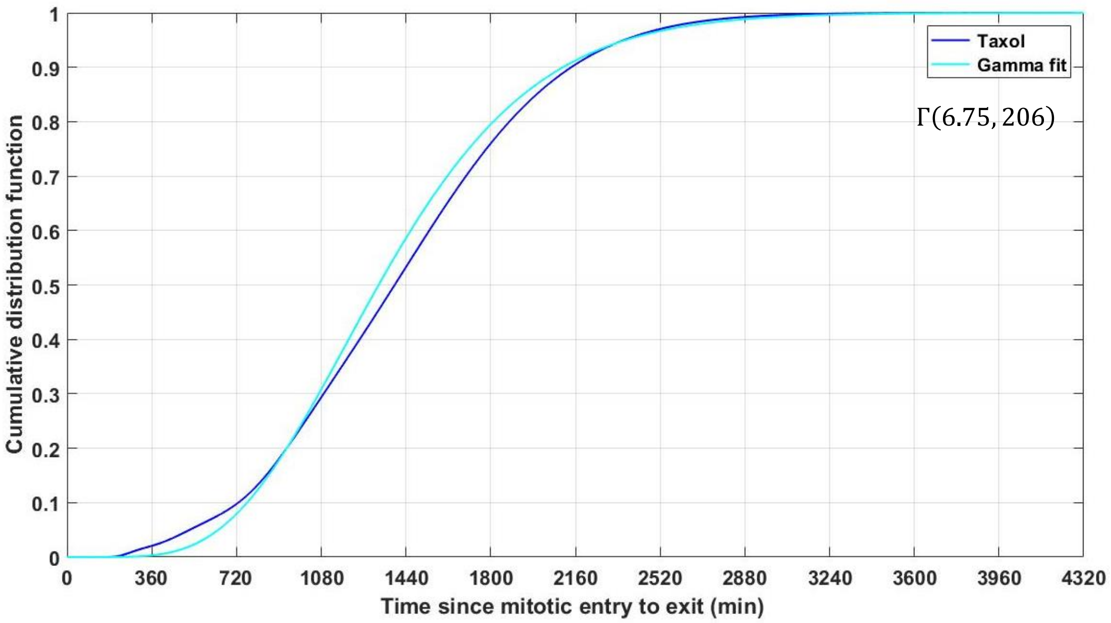
Taxol
Gamma fit
Γ(6.75, 206)
Cumulative distribution function
Time since mitotic entry to exit (min)
0
0.1
0.2
0.3
0.4
0.5
0.6
0.7
0.8
0.9
1
0
360
720
1080
1440
1800
2160
2520
2880
3240
3600
3960
4320

I

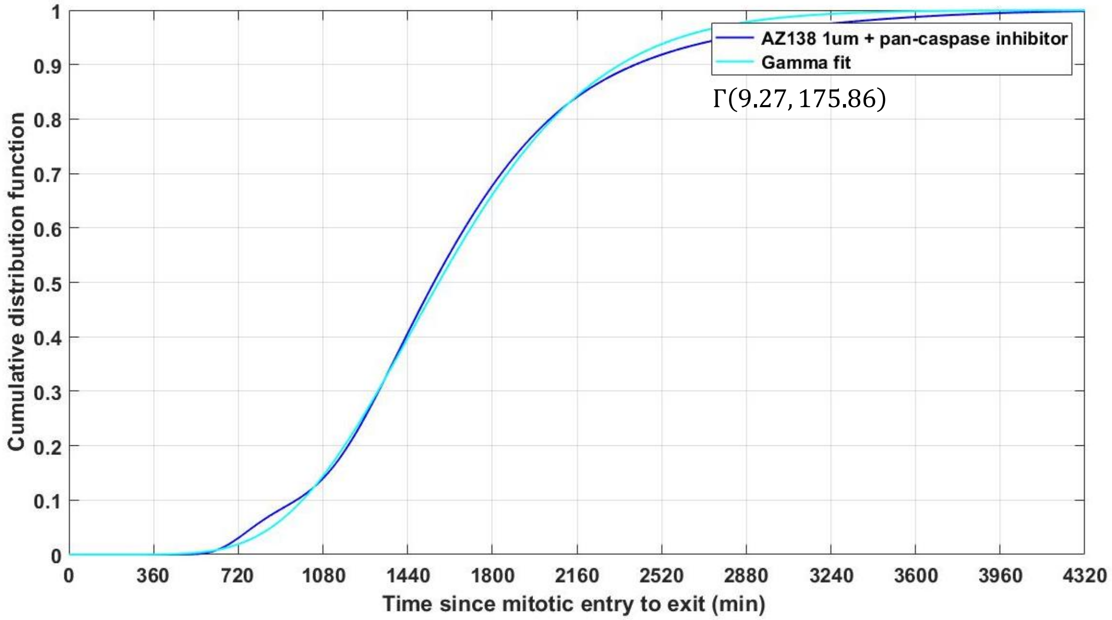

AZ138 1um + pan-caspase inhibitor
Gamma fit
Γ(9.27, 175.86)
Cumulative distribution function
0
0.1
0.2
0.3
0.4
0.5
0.6
0.7
0.8
0.9
1
0
360
720
1080
1440
1800
2160
2520
2880
3240
3600
3960
4320
Time since mitotic entry to exit (min)

J

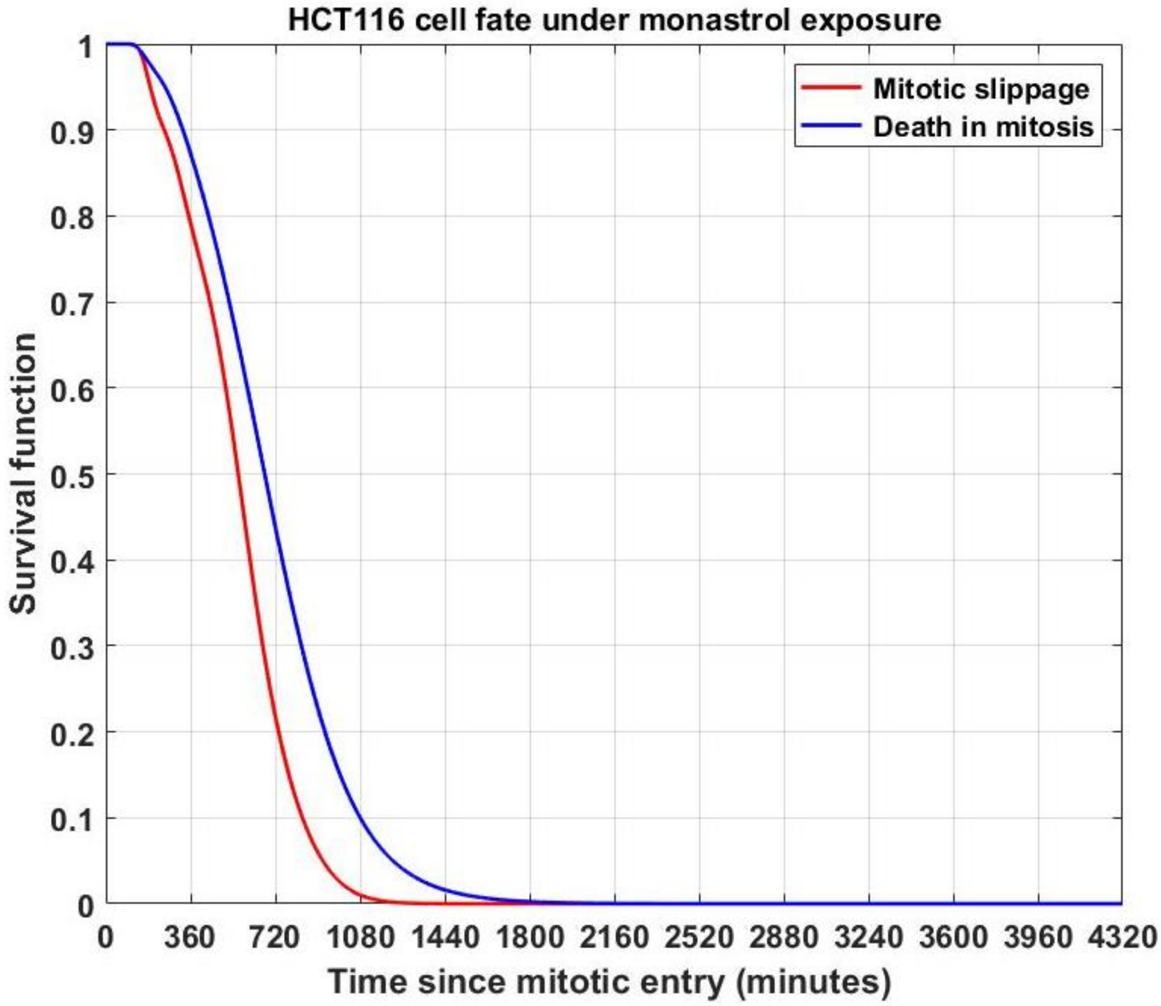

HCT116 cell fate under monastrol exposure
Mitotic slippage
Death in mitosis
Survival function
0
0.1
0.2
0.3
0.4
0.5
0.6
0.7
0.8
0.9
1
0
360
720
1080
1440
1800
2160
2520
2880
3240
3600
3960
4320
Time since mitotic entry (minutes)

K

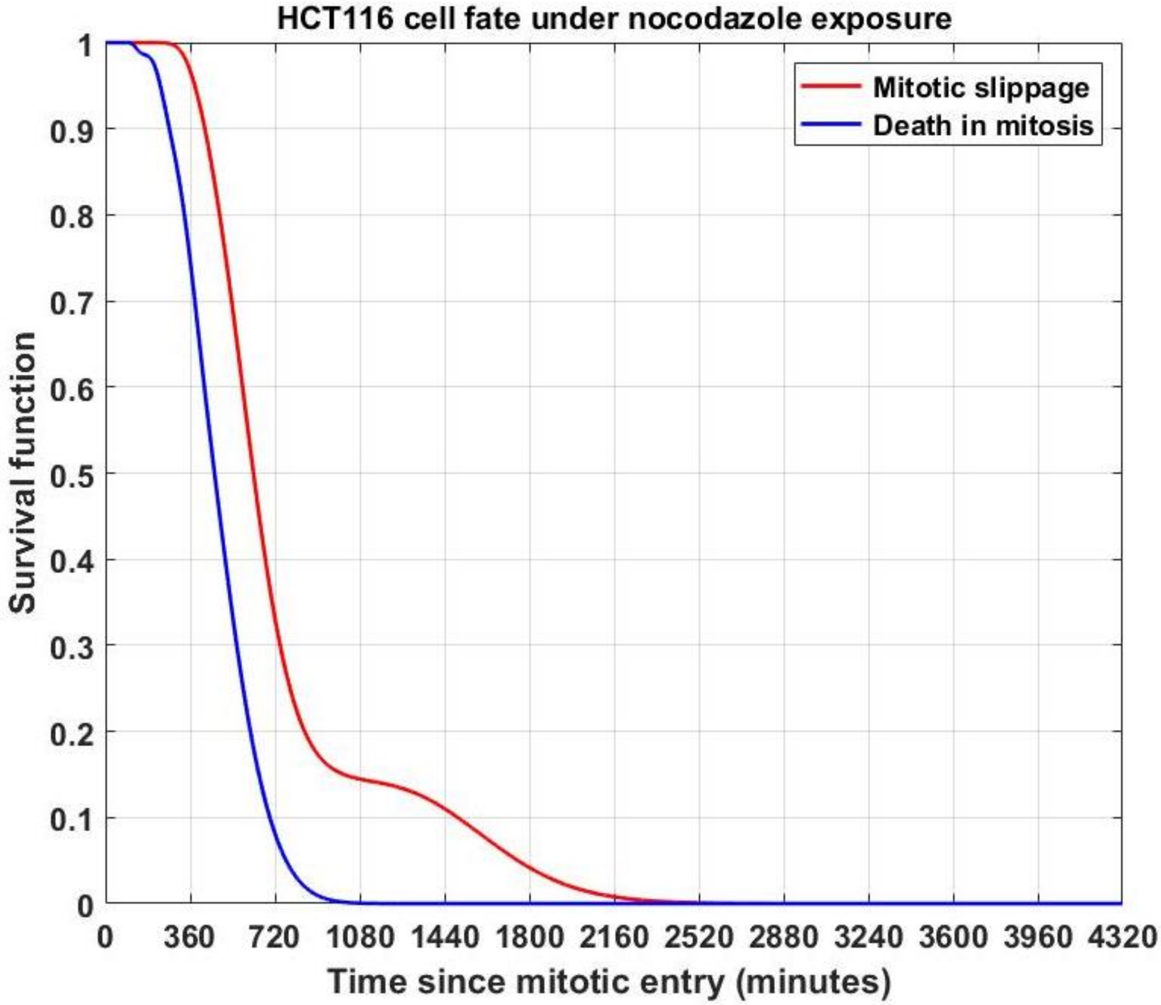
HCT116 cell fate under nocodazole exposure
Mitotic slippage
Death in mitosis
Survival function
1
0.9
0.8
0.7
0.6
0.5
0.4
0.3
0.2
0.1
0
0
360
720
1080
1440
1800
2160
2520
2880
3240
3600
3960
4320
Time since mitotic entry (minutes)

L

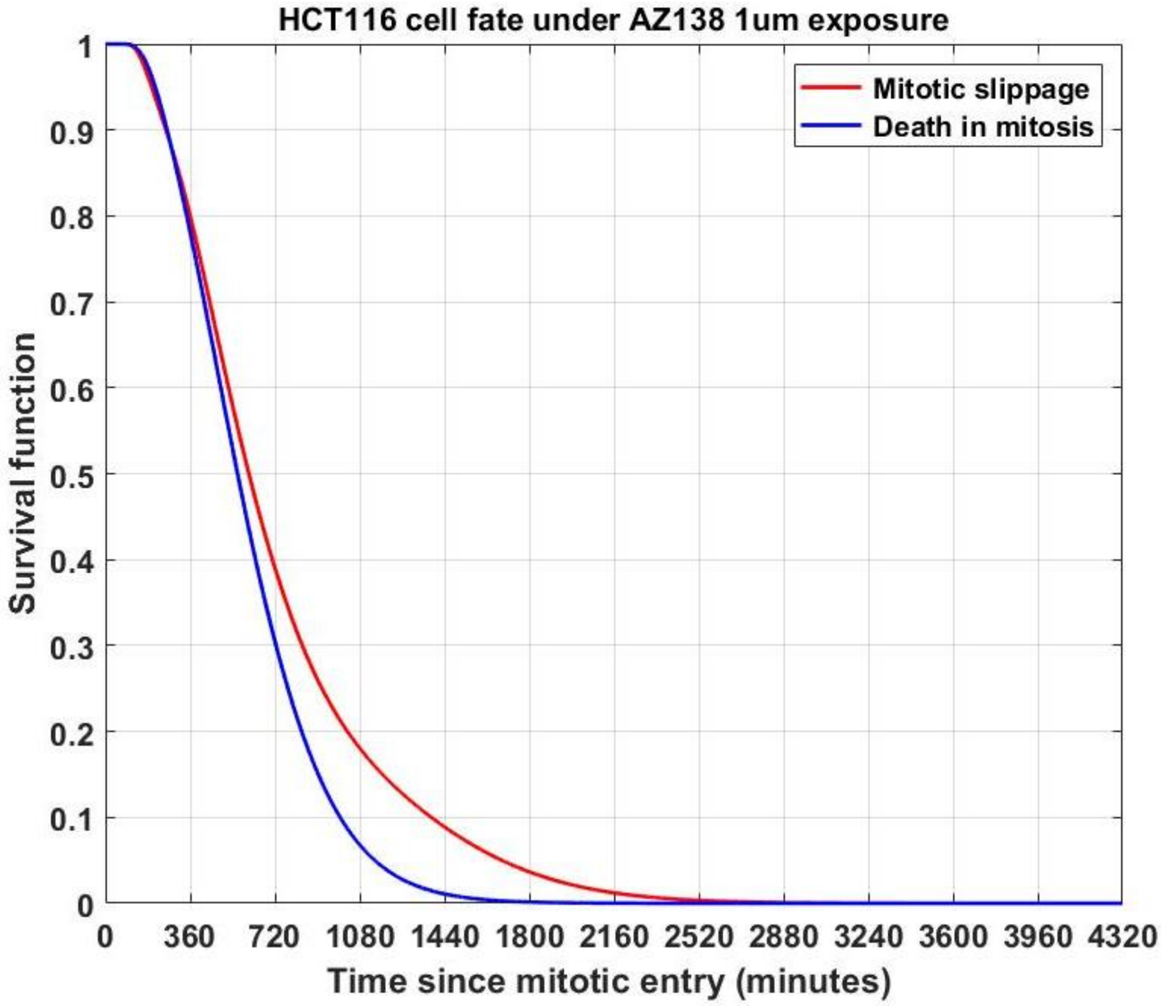
HCT116 cell fate under AZ138 1um exposure
Mitotic slippage
Death in mitosis
Survival function
1
0.9
0.8
0.7
0.6
0.5
0.4
0.3
0.2
0.1
0
0 360 720 1080 1440 1800 2160 2520 2880 3240 3600 3960 4320
Time since mitotic entry (minutes)

M

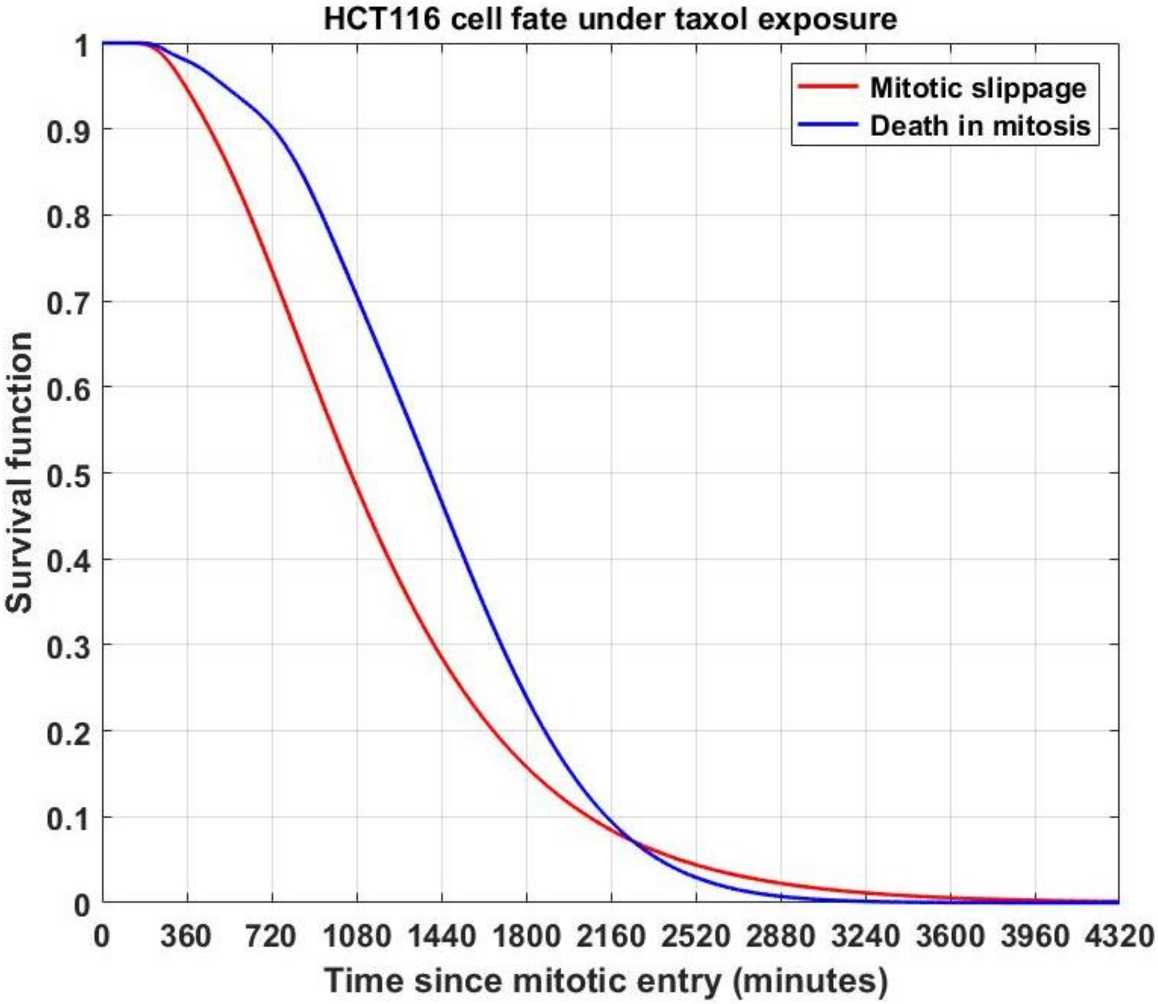
HCT116 cell fate under taxol exposure
Mitotic slippage
Death in mitosis
Survival function
Time since mitotic entry (minutes)
0
360
720
1080
1440
1800
2160
2520
2880
3240
3600
3960
4320
0
0.1
0.2
0.3
0.4
0.5
0.6
0.7
0.8
0.9
1

N

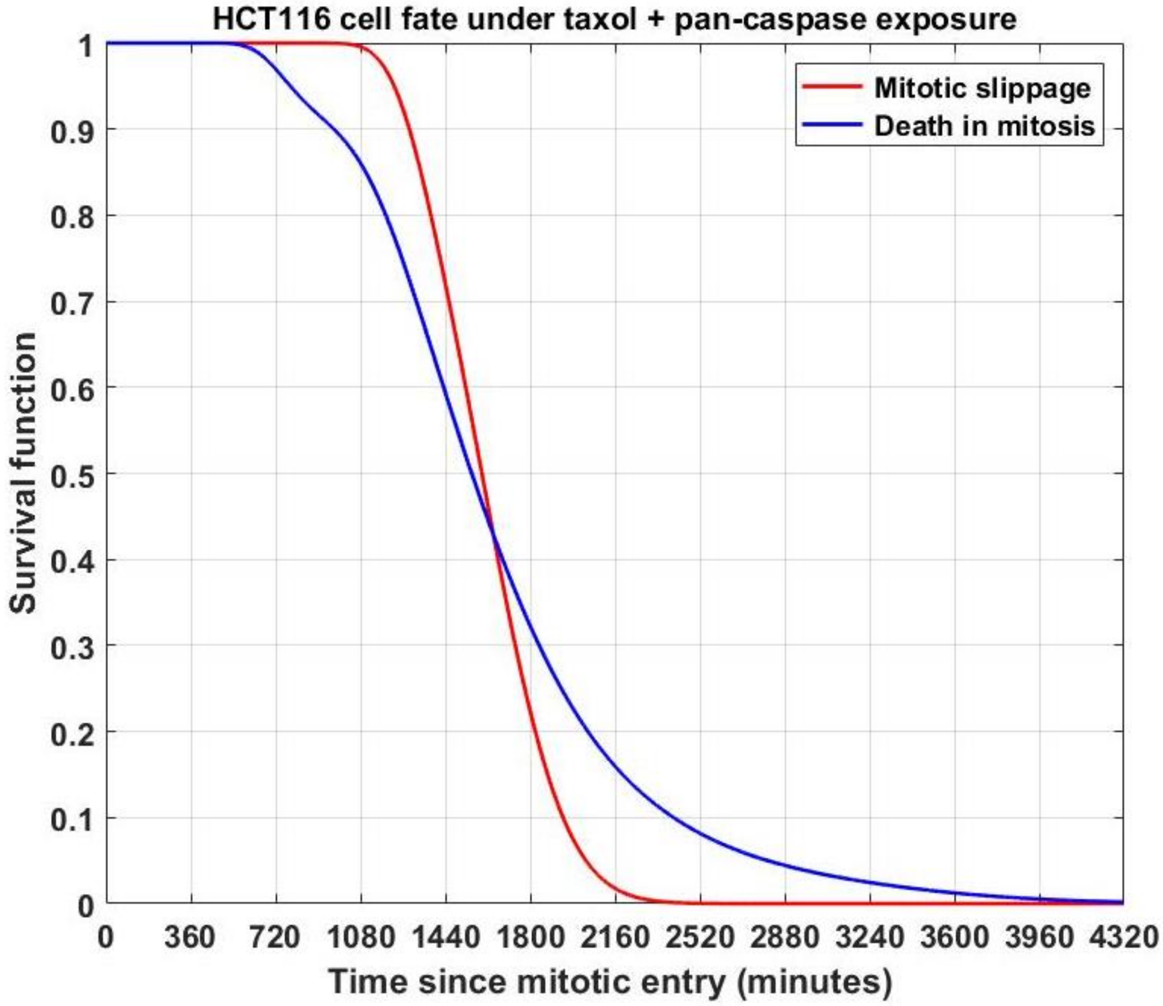

Supplementary Figure 5. **HCT116 cell death and slippage responses across drugs can be characterized by Gamma distributions even in the presence of the pan-caspase inhibitor Boc-D-FMK.**

The cumulative distribution functions (CDF) for the death in mitosis and mitotic slippage show the fraction of RKO cells that either died or slipped after entering mitosis as a function of time. Data are adapted from the experimental findings reported in Figure S5A in [1] in the presence of the pan-caspase inhibitor Boc-D-FMK. Therein, the HCT116 cellular fate following prolonged exposure to four different drugs (monastrol, nocodazole, taxol and AZ138) was measured, based on the duration of drug-induced mitotic arrest. Cell death or slippage responses across drugs in panels (A) – (I) can be characterized by the cell-cycle age "a"-dependent Gamma distribution $\Gamma(a; k, \theta)$, with shape parameter

k and scale parameter θ. For each panel, we report the corresponding Gamma CDF parameters in the inset adjacent to the figure legend. We additionally report the HCT116 cell fate in the death in mitosis and mitotic slippage pathways as CDFs under (J) monastrol, (K) 30ng/ml nocodazole, (L) 1 μM AZ138, (M) 0.1 μM taxol and (N) 0.1 μM taxol, + 100 μM Boc-D-FMK (a pan-caspase inhibitor) prolonged exposure.

Bibliography

1. Gascoigne, K.E., Taylor, S.S. Cancer cells display profound intra- and interline variation following prolonged exposure to antimitotic drugs. *Cancer Cell.* **14(2)**, 111-122 (2008).
